# TOG Proteins Are Spatially Regulated by Rac-GSK3β to Control Interphase Microtubule Dynamics

**DOI:** 10.1371/journal.pone.0138966

**Published:** 2015-09-25

**Authors:** Kathryn P. Trogden, Stephen L. Rogers

**Affiliations:** 1 Department of Biology, University of North Carolina at Chapel Hill, Chapel Hill, NC 27599, United States of America; 2 Carolina Center for Genome Sciences, University of North Carolina at Chapel Hill, Chapel Hill, NC, 27599, United States of America; 3 Lineberger Comprehensive Cancer Center, University of North Carolina at Chapel Hill, Chapel Hill, NC, 27599, United States of America; University of Illinois at Chicago, UNITED STATES

## Abstract

Microtubules are regulated by a diverse set of proteins that localize to microtubule plus ends (+TIPs) where they regulate dynamic instability and mediate interactions with the cell cortex, actin filaments, and organelles. Although individual +TIPs have been studied in depth and we understand their basic contributions to microtubule dynamics, there is a growing body of evidence that these proteins exhibit cross-talk and likely function to collectively integrate microtubule behavior and upstream signaling pathways. In this study, we have identified a novel protein-protein interaction between the XMAP215 homologue in *Drosophila*, Mini spindles (Msps), and the CLASP homologue, Orbit. These proteins have been shown to promote and suppress microtubule dynamics, respectively. We show that microtubule dynamics are regionally controlled in cells by Rac acting to suppress GSK3β in the peripheral lamellae/lamellipodium. Phosphorylation of Orbit by GSK3β triggers a relocalization of Msps from the microtubule plus end to the lattice. Mutation of the Msps-Orbit binding site revealed that this interaction is required for regulating microtubule dynamic instability in the cell periphery. Based on our findings, we propose that Msps is a novel Rac effector that acts, in partnership with Orbit, to regionally regulate microtubule dynamics.

## Introduction

Microtubules interact with the small GTPase Rac in a complex pattern of cross-talk at the leading edge of motile cells. Growing microtubules induce cortical Rac activation by locally activating a guanine exchange factor (GEF) to induce protrusion and directional migration [1]. In what is thought to be a positive feedback loop, active Rac promotes persistent microtubule growth in the lamellae and lamellipodia by locally regulating the activity of microtubule-associated proteins (MAPs) [2]. At least three MAPs have been implicated in regulation of microtubule dynamics downstream of Rac. The first is CLIP-170, a microtubule plus end interacting protein (+TIP) that interacts with the Rac effector IQGAP1 to capture microtubule plus ends at the plasma membrane [3]. The second is stathmin/OP18, a microtubule destabilizing factor that is locally inhibited at the leading edge due to phosphorylation by the Rac effector kinase Pak [4]. The third is CLASP, another +TIP that suppresses dynamics, leading to increased stabilization of microtubules at the leading edge of polarized fibroblasts [5,6]. CLASP binds directly to microtubules through a central lattice-binding domain and localizes to growing plus ends through an interaction with EB1 [7]. In the cell cortex, CLASP is phosphorylated by GSK3β, which blocks its ability to bind to the microtubule lattice, thus targeting it to growing plus ends [8]. At the leading edge, GSK3β is locally inhibited by Rac and dephosphorylated CLASP binds along the microtubule lattice [7]. Although local inhibition of stathmin and activation of CLASP seem to be necessary for persistent microtubule growth at the leading edge, neither factor is sufficient, suggesting that other regulatory mechanisms remain to be discovered.

The XMAP215/Dis1 proteins are a family of conserved +TIPs that have not been extensively studied in the context of cell polarity. XMAP215 was originally discovered as a factor from *Xenopus* extracts that potently promoted microtubule growth from the plus end [9]. This family is conserved across all eukaryotic taxa and, where studied, homologues have been shown to be important for promoting microtubule dynamics [10, 11]. The XMAP215 family has a conserved domain structure of five arrayed NH_2_-terminal TOG domains. Crystal structures of these domains have revealed them to be flat and “paddle”-like consisting of six tandem HEAT repeats [12,13]. TOG domains are proposed to bind tubulin using the loop regions between helices of the HEAT repeats on one face [14]. The number of tubulin dimers that can be bound by individual TOG domains is not clear [15,16]. High-resolution in vitro assays have suggested two possible mechanisms for XMAP215 activity in promoting microtubule polymerization. One study found that XMAP215 was able to bind to growing microtubule plus ends and processively add individual tubulin dimers [17]. Another study found that XMAP215 may template the assembly of tubulin oligomers, which are then added to the plus end of the microtubule [18].

XMAP215 family members also exhibit microtubule depolymerizing activity in vitro. They depolymerize GMPCPP microtubules, a slowly hydrolysable form that mimics the GTP or GDP-Pi bound state [17,19]. The depolymerizing activity is thought to be the reverse of its polymerizing reaction, where it removes tubulin dimers from the plus-end using its TOG domains [17]. This depolymerizing activity seems to be relevant in cells, as depletion of the *Drosophila* homologue Mini-spindles (Msps) causes microtubules to become non-dynamic and spend more time in the pause state [20,21]. This dual mechanism is thought to increase the dynamics of microtubules in cells, catalyzing the transition from the pause state to growth or shrinkage [21].

In this study, we used cultured *Drosophila* S2 cells as a model to study the role of Msps in regulating microtubule dynamics downstream of Rac. We found that Rac and the kinase GSK3β regulate Msps localization and dynamics in S2 cells. Regulation of Msps is indirect and occurs through phosphorylation of the *Drosophila* CLASP homologue, Orbit/MAST. Our data suggest that GSK3β phosphorylates Orbit thereby preventing its interaction with Msps. This protein-protein interaction causes recruitment of Msps to the microtubule lattice, distal from the plus end. This relocalization of Msps is required for proper regulation of microtubule dynamics; when the Msps-Orbit interaction site is ablated by mutation, microtubules spend most of their time growing and exhibit decreased time spent in shrinkage and pause. Our results identify Msps as a key regulator of microtubule dynamics downstream of Rac and reveal a novel protein-protein interaction between +TIPs that is required for normal dynamic instability.

## Materials and Methods

### Cell Culture and RNA Interference

Culture and RNAi of *Drosophila* S2 cells were performed as previously described [22]. S2 cells (*Drosophila* Genomics Resource Center, Bloomington, IN) were cultured in SF900II medium supplemented with 100x antibiotic-antimycotic (Invitrogen, Carlsbad, CA). For RNAi, the T7 promoter sequence was appended to gene-specific primer sequences to generate dsRNA using T7 RiboMAX in vitro transcription (Promega, Madison, WI). Primer sequences for NT Orbit dsRNA are as follows: forward, 5’ AGGTACAGCTCGCCGAGGATCTGGTGACATTCCTTA 3’ and reverse, 5’ GCCTCCCTCACATTAACTGTGGGATCTCCGAGAA 3’. CT Orbit dsRNA primer sequences were as follows: forward, 5’ TACGGACGTGGTCATCGCTGGCCTGC 3’ and reverse, 5’ GCAGGCCAGCGATGACCACGTCCGTA. Primers for other dsRNAs were made as previously described: SK (control) [22], Rho1, Cdc42, Rac and Mtl [23], GSK3β [24], EB1, Msps and 5’UTR Msps [21], Sentin [25], Orbit and 5’UTR Orbit [26].

### Immunofluorescence and Live-Cell Imaging

Fixation of S2 cells was adapted from previously described protocols [22]. S2 cells were plated onto 0.5mg/ml concanavalin A (ConA)-treated glass coverslips for 1 hour and fixed with cold methanol. Antibodies used in this study include: phosphohistone H3 (Cell Signaling Technology, Beverly, MA), Msps [21] 1:1000, Orbit [26] 1:500, Sentin [27], EB1 [28] and α-tubulin (DM1α; Sigma-Aldrich, St. Louis, MO) 1:500. Secondary antibodies include Cy2, Rhodamine red and Cy5 (Jackson ImmunoResearch Laboratories, West Grove, PA) used at a concentration of 1:200. Actin was stained using Alexa Fluor 488 phalloidin (Invitrogen). Cells were imaged on an Eclipse Ti-E microscope with a 100x oil NA-1.45 objective, driven by NIS Elements software. Images were captured with a cooled charge-coupled device camera (CoolSNAP HQ, Roper Scientific). For live-cell imaging, 0.5mg/ml ConA-coated coverslips attached to drilled 35-mm tissue culture dishes with UV-curable adhesive (Norland Products Cranbury, NJ) were used. Cells were seeded in Schneider’s *Drosophila* medium supplemented with 10% fetal bovine serum and 100x antibiotic-antimycotic (Invitrogen) and allowed to attach for 1 hr. Time-lapse imaging was performed on a motorized total internal reflection fluorescence (TIRF) system (Nikon) mounted on an inverted microscope (Ti; Nikon) equipped with a 100x/1.49 objective lens, driven by NIS Elements software. Images were captured with an Andor-Clara Interline camera (Andor Technology, Belfast, UK). All images were processed using Photoshop (CS version 14.2; Adobe Systems, Mountain View, CA).

### Immunoprecipitation/Western Blotting

S2 cells after 7 days of RNAi were lysed in cell lysis buffer (CLB; 50mM Tris, 150mM NaCl, 0.5mM EDTA, 1mM dithiothreitol, 0.5% Triton X-100 and 2.5mM phenylmethylsulfonyl fluoride) and precleared and diluted two-fold with CLB. Samples were removed for input controls before mixing with α-Msps for 2 hours at 4°C, then protein A-Sepharose beads (Sigma-Aldrich) were incubated with the antibody-lysate mixture for 2h at 4°C. Beads were washed three times with 0.5ml CLB and boiled in SDS-PAGE sample buffer. GFP IPs were performed as previously described [29]. Proteins were analyzed with 15% SDS PAGE gels. GSK 3β RNAi efficacy was assayed by immunoblot for Armadillo and protein loads were normalized using α-tubulin antibody (Sigma-Aldrich). Additional antibodies include: α-Msps [21], α-Orbit [26] and α-Armadillo (Developmental Hybridoma Bank).

### Molecular Biology and Transfection

Expression constructs for full length EB1 and Msps were made as previously described [21]. Full length Sentin GFP was subcloned using the Gateway TopoD pEntr system (Invitrogen) into a final zeocin-selectable pIZ backbone (Invitrogen) that had both the metallothionein promoter and the Gateway (Invitrogen) LR recombination sites inserted into the multicloning site. Full length GFP Orbit was subcloned using the Gateway TopoD pEntr system (Invitrogen) into a final ampicillin-selectable metallothionein promoter pMT backbone (Invitrogen). Fragments of Orbit were subcloned into a metallothionein promoter, pMT A vector backbone (Invitrogen) that contained a COOH-terminal fusion of GFP. Fragments of Msps were subcloned into a metallothionein promoter, pMT A vector backbone (Invitrogen) that contained a COOH-terminal fusion of GFP or mCherry. α-tubulin was expressed in a dual expression vector under the TUB84 promoter [30], a second site with a metallothionein promoter, pMTB was subcloned with either Rac1V12, Cdc42V12, Rho1V14 or GSK3βS9A. Mutants were made using PCR-based site directed mutagenesis. Transfections were performed with the Amaxa Nucleofector II transfection system (Lonza, Basel, Switzerland) or with FuGENE HD (Promega, Madison, WI) according to manufacturers’ protocol. The constructs were induced 24 hours after transfection with 100uM CuSO_4_ for 12–18 hours.

### Colocalization Analysis

Colocalization was measured using the Manders’ coefficient as previously described [31,32] with additional processing prior to analysis. To minimize background, the subtract background function in ImageJ (National Institutes of Health [NIH], Bethesda, MD) using a rolling ball radius of 50 pixels, followed by use of the Despeckle function. Statistical anaylsis was performed using a one-way ANOVA, statistically significant differences from the control are denoted in the graphs were *** p<0.0001.

### Microtubule, EB1 and Actin Retrograde Flow Measurements

Actin retrograde flow was measured using GFP-Actin expressing cells. Movies were taken for 4 minutes with 1 second intervals. Kymographs of the actin in the periphery were made and the slope actin particles was used to find the flow rate at um/min. Movies of microtubules were taken at 1 second intervals for 4 minutes. The microtubules were then tracked using the ImageJ plug in MTrackJ [33]. The x and y coordinates were then placed in a custom algorithm coded in Visual Basic using Excel generously provided by Dr. Dan Buster, University of Arizona. Diamond graphs were created as previously described [34] and were jointly normalized. EB1 comets were tracked using the Manual Tracking plug in from ImageJ. Comets were tracked for at least 10 frames (30 seconds). Movies were taken for 5 minutes with 3-second intervals to allow for dual color imaging and EB1 and Msps.

## Results

### *Drosophila* S2 Cells Possess Two Populations of Microtubules

*Drosophila* S2 cells attach to concanavalin A-treated coverslips and assemble prominent lamellae and lamellipodia in a Rac-dependent manner [28]. As they spread, microtubules partition into two populations: cell cortex and peripheral microtubules. Cell cortex microtubules occupy the central organelle-rich region of the cells, often display a prominent curvature, and are difficult to track due to crowding (S1 Movie). Peripheral microtubules grow into the lamellae, often align perpendicularly to the cell margin, and exhibit a stereotypical pattern of dynamics in which they grow out to the edge of the cell, briefly pause and shrink back into the cell interior (S1 Movie). Peripheral microtubules polymerize against the centripetal force of actin retrograde flow and can often appear to be in a state of pause when actually growing at an equivalent rate to actin. In this respect, peripheral microtubules in S2 cells resemble "pioneer" microtubules described at the leading edge of motile cells [2]. These different populations of microtubules exhibit significant differences in their growth rates as well. Tracking of EB1 comets revealed that cell cortex microtubules grow at 12.75 ± 2.44 um/min, while those in at the periphery grow slower at 10.04 ± 1.88 um/min indicating that they may have different dynamics (S1A and S1B Fig).

### Rac and GSK3β Regulate Orbit and Msps Localization in S2 Cells

**Fig 1:**
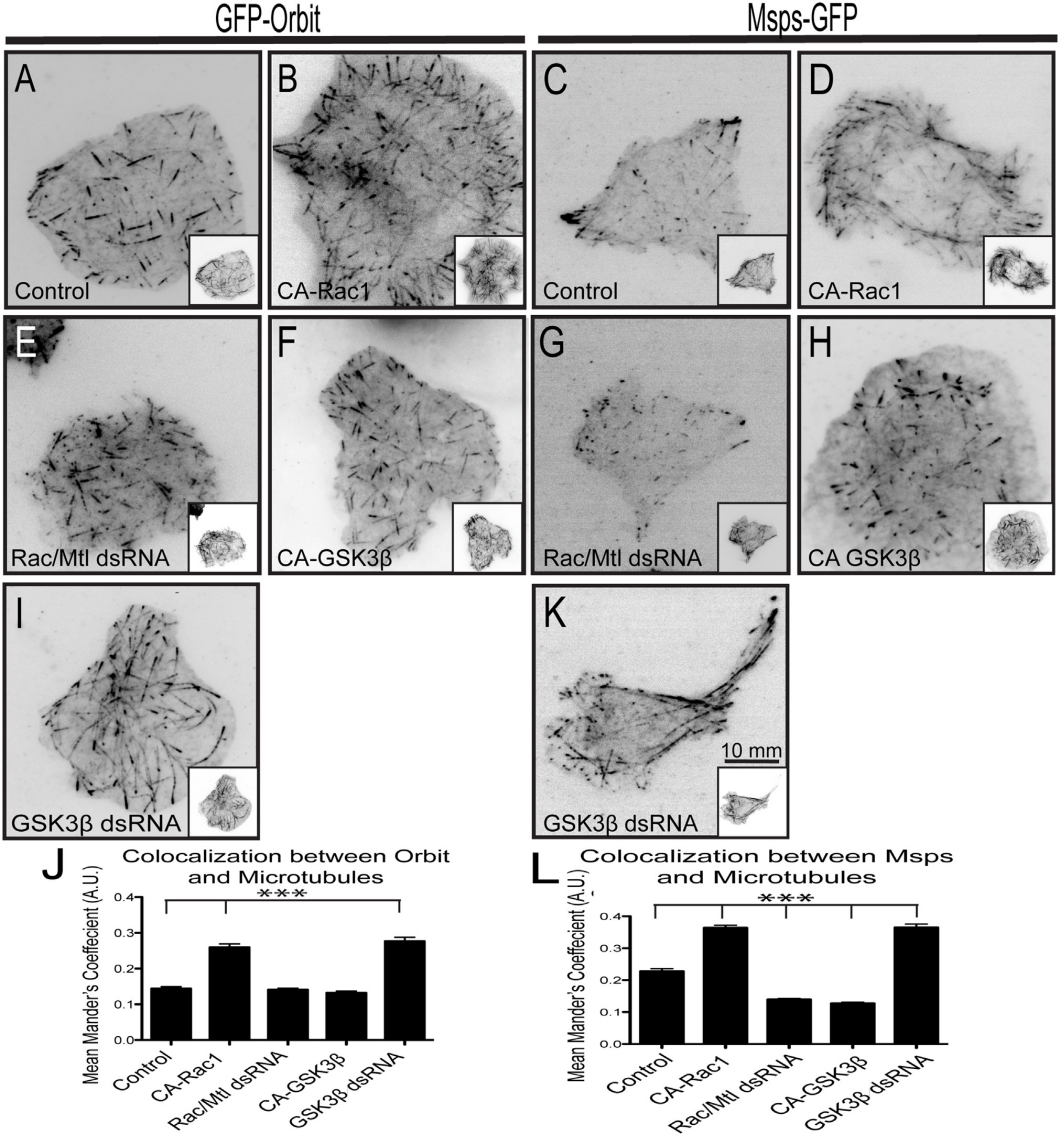
Orbit and Msps localization is regulated by Rac-GSK3β signaling. GFP-Orbit was expressed in cells with a dual expression vector containing tRFP-α-tubulin alone (A) with CA-Rac1 (B) or with CA-GSK3β (F). Rac1/Rac2/Mtl (E) or GSK3β (I) were knocked down with dsRNA in a cell expressing tRFP-α-tubulin alone. (C) Msps-GFP is expressed in cells with a dual expression vector containing tRFP-α-tubulin alone (C) with CA-Rac1 (D) or with CA-GSK3β (H). Rac1/Rac2/Mtl (G) or GSK3β (K) were knocked down with dsRNA in cell expressing tRFP-α-tubulin alone. Tubulin images are shown as insets. Changes in the co-localization of Orbit (J) and Msps (L) with microtubules were measured using the Mander’s coefficient. An increase indicates increased lattice binding and a decrease indicates decreased lattice binding. N = 90 cells per condition for quantification from three experiments *** p<0.0001.

Based on previous work showing that CLASP regulates microtubule dynamics in the lamellae of mammalian cells, we asked if the *Drosophila* homologue Orbit/MAST is also regulated by Rac and GSK3β in S2 cells. We confirmed previous studies that Orbit localizes exclusively to comet structures at microtubule plus-ends in interphase cells and never exhibited lattice association in control cells (Fig 1A, S1E Fig). However, when S2 cells were transfected with a constitutively active Rac1 mutant (CA-Rac1) or GSK3β is depleted with RNAi, Orbit binds to the lattice throughout the cell, leading to increased co-localization with tubulin (Fig 1B and 1I, S1F and S1K Fig, knock down efficiency was confirmed by scoring for mitotic defects S1U Fig). We noted that expression of CA-Rac1 alone had a dramatic effect on microtubule organization (S1C and S1D Fig), causing a decrease in cell cortex microtubules and an increase in the periphery, however, the exact mechanism of this effect is not known. This change in lattice binding was measured as colocalization between Orbit and microtubules, using the Mander’s coefficient [31,32] (a scale of zero to one, with zero representing no colocalization; Fig 1J and S1O Fig). To perform this quantification we processed the images by subtracting background cytoplasmic fluorescence and filtered the images to reduce noise (S2A–S2J Fig). As a negative control we determined the Mander's coefficient between microtubules and GFP-actin; following image corrections the peripheral actin network minimally colocalizes with tubulin producing a mean Mander’s coefficient of 0.06 ± 0.03 (S2A–2E and S2K Fig). As a positive control, we measured colocalization between microtubules and murine GFP-MAP2C, a neuronal MAP that decorated microtubules along their lengths and yielded a Mander’s coefficient of 0.8 ± 0.09 (S2F–S2K Fig). We also performed a control to determine if the number of EB1 comets per cell impacted our colocalization by Mander's coefficient in control cells and in cells expressing CA-Rac1; plotting the number of EB1 comets versus Mander's coefficient revealed that the number of microtubule plus ends does not affect our colocalization analysis (S2L Fig). These controls demonstrate that the Mander’s coefficient accurately reflects changes in the localization of +TIPs. Simultaneous depletion of all three Rac proteins (Rac1, Rac2, and Mtl) or overexpression of constitutively active GSK3β (CA-GSK3β) had no effect on Orbit localization to microtubule plus ends (Fig 1E, 1F and 1J, S1J and S1O Fig). These data suggest that *Drosophila* Orbit can be regulated by Rac and GSK3β.

We previously described the dynamics of Msps in living cells and found that it exhibits a similar localization to that described for CLASP in mammalian cells: Msps binds to the lattice in the lamellae of S2 cells and tip tracks in the cell cortex [7,21,23] (Fig 1C, S1G Fig).

We wanted to determine if Msps’ localization on the microtubule correlated with a change in the rate of microtubule growth. We tracked EB1-GFP comets and observed that they were fastest in the cell interior (12.75 ± 2.44 um/min, purple box) and slower at the periphery (10.04 ± 1.88 um/min, green box) (S1A and S1B Fig). This decrease in peripheral growth rates is likely due, at least in part, to antagonistic interactions between microtubules and the lamellipodial actin network undergoing retrograde flow at a rate of 2.27 ± 0.39 um/min (S4D Fig). Interestingly EB1 comets in regions of the cell where Msps is bound to the lattice exhibit statistically significant slower rates of growth (9.36 ± 1.88 um/min, blue box) (S1A and S1B Fig). Thus, S2 cells possess three populations of microtubules that differ in their growth velocities: cell cortex microtubules, peripheral microtubules, and peripheral microtubules with lattice-associated Msps.

To test the hypothesis that the Rac-GSK3β pathway locally regulates the interaction between Msps and microtubules we expressed CA-Rac1 or depleted GSK3β using RNAi. Both of these treatments triggered recruitment of Msps to the microtubule lattice throughout the cells (Fig 1D, 1K and 1L, S1H, S1M and S1N Fig). Conversely, expression of CA-GSK3β or knock-down of Rac1/Rac2/Mtl caused a loss of Msps on the lattice in the periphery of the cell (Fig 1G, 1H and 1L, S1L and S1N Fig). Changes in the levels/activation of the other Rho GTP family members Cdc42 and Rho did not affect Msps localization, indicating that this pathway is specific for Rac (S1P–S1T Fig). These results suggest that *Drosophila* Orbit and Msps are both regulated by the Rac-GSK3β signaling axis.

### Msps Is Regulated by GSK3β through Orbit

**Fig 2:**
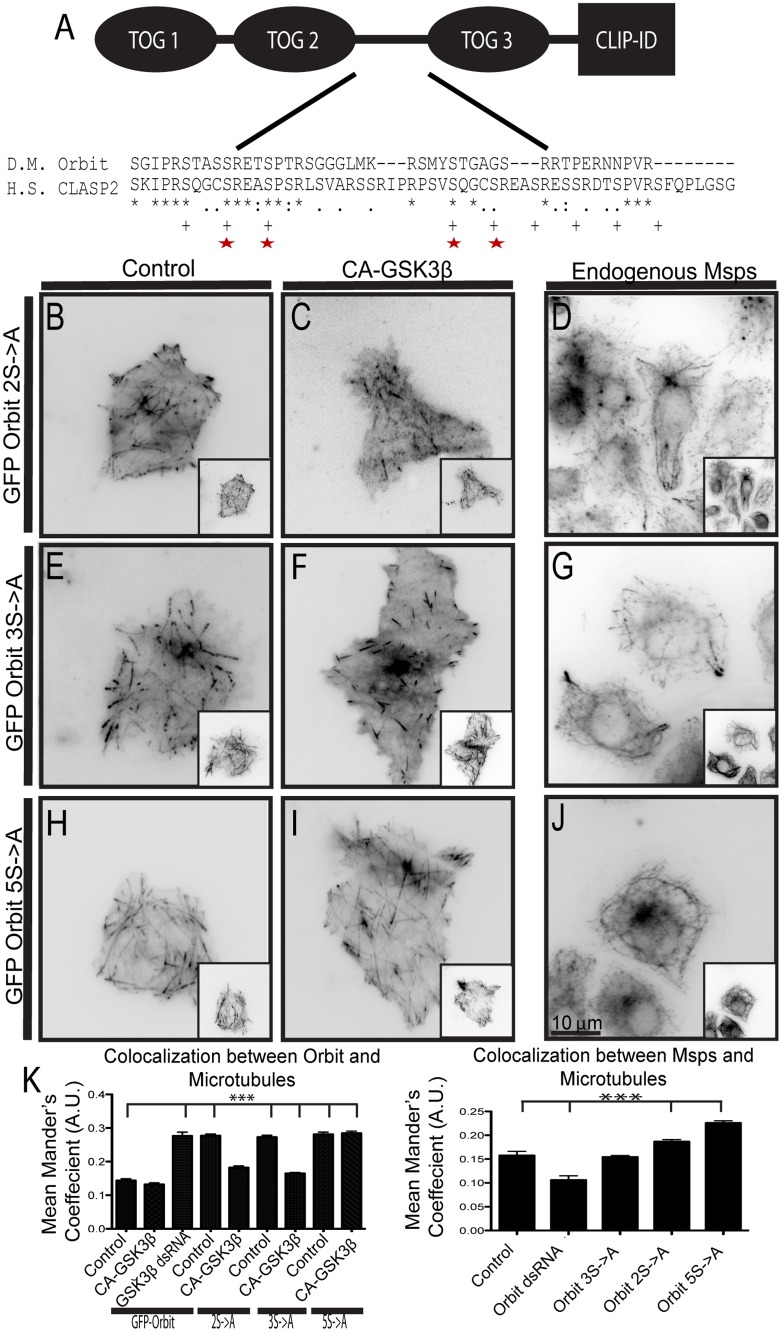
Orbit is phosphorylated by GSK3β in the linker region between TOG2 and TOG3. (A) Domain structure of Orbit with a Clustal alignment of the GSK3β phosphorylation region. Serines phosphorylated in *Homo sapiens* (H.S.) CLASP2 denoted by plus marks (+), serines conserved in *Drosophila Melanogaster* (D.M.) Orbit denoted by red stars. (B-C) GFP-Orbit 2S->A was expressed in cells with a dual expression vector containing tRFP-α-tubulin alone (B) or with CA-GSK3β (C). (D) Endogenous Msps and α-tubulin were stained in cells transfected with 2S->A. (E-F) GFP-Orbit 3S->A is expressed in cells with a dual expression vector containing α-tubulin-tRFP alone (E) or with CA-GSK3β (F). (G) Endogenous Msps and α-tubulin were stained in cells transfected with 2S->A. (H-I) GFP-Orbit 5S->A was expressed in cells with a dual expression vector containing tRFP-α-tubulin alone (H) or with CA-GSK3β (I). (J) Endogenous Msps and α-tubulin were stained in cells transfected with 5S->A. Tubulin images are shown as insets. (K) Changes in co-localization of Orbit (left) and endogenous Msps (right) were measured using the Mander’s coefficient, n = 90 cells from two (endogenous Msps) or three (GFP-Orbit) experiments. *** p<0.0001.

Our results that Rac and GSK3β govern Msps-microtubule interactions represent the first demonstration that an XMAP215 family member is regulated during interphase. Msps does not contain a GSK3β consensus sequence (Ser/Thr—X—X—X-Ser/Thr-P) and we were unable to detect a shift in Msps mobility by SDS-PAGE following inhibition or overexpression of GSK3β (data not shown). However, Orbit has a GSK3β consensus sequence in the linker region between TOG domains 2 and 3 that is conserved with the GSK3β phosphorylation site in CLASP (Fig 2A, phosphorylated serines indicated by plus signs (+)). To test the hypothesis that GSK3β regulates Msps localization indirectly through an interaction with Orbit we mutated the five conserved serines in the GSK3β consensus site to alanines to make a non-phosphorylatable Orbit mutant (Fig 2A, indicated by stars). Mutation of the first three serines (3S->A) had a mild effect on the localization of Orbit, as was described for CLASP [8]. In control cells 3S->A bound along the lattice of the microtubule, however, coexpression with CA-GSK3β prevented lattice binding (Fig 2E, 2F and 2K). We also expressed 3S->A in S2 cells, fixed, and stained for endogenous Msps and did not observe a change in its localization pattern (Fig 2G and 2K). However, mutation of the second two conserved serines had a greater effect on Orbit localization; this mutant (2S->A) colocalized more with microtubules in control cells and was modestly displaced from the lattice by co-expression of CA-GSK3β (Fig 2B, 2C and 2K). In addition, the 2S->A mutant recruited endogenous Msps to the lattice (compare to S1G Fig; Fig 2D and 2K). Mutation of all five conserved serines had the greatest effect; this mutant bound to the lattice even when co-expressed with CA-GSK3β and its expression caused endogenous Msps to bind along the lattice, as well (Fig 2H–2K). All of these experiments were conducted on cells depleted of endogenous Orbit using RNAi. We also generated phosphomimetic mutants of Orbit, discussed below (S2 Fig). Our results indicate that GSK3β phosphorylates Orbit on five conserved serine residues and that when these five serines are mutated to alanines Orbit becomes insensitive to CA-GSK3β expression, binds along the microtubule lattice, and recruits Msps there, as well.

**Fig 3:**
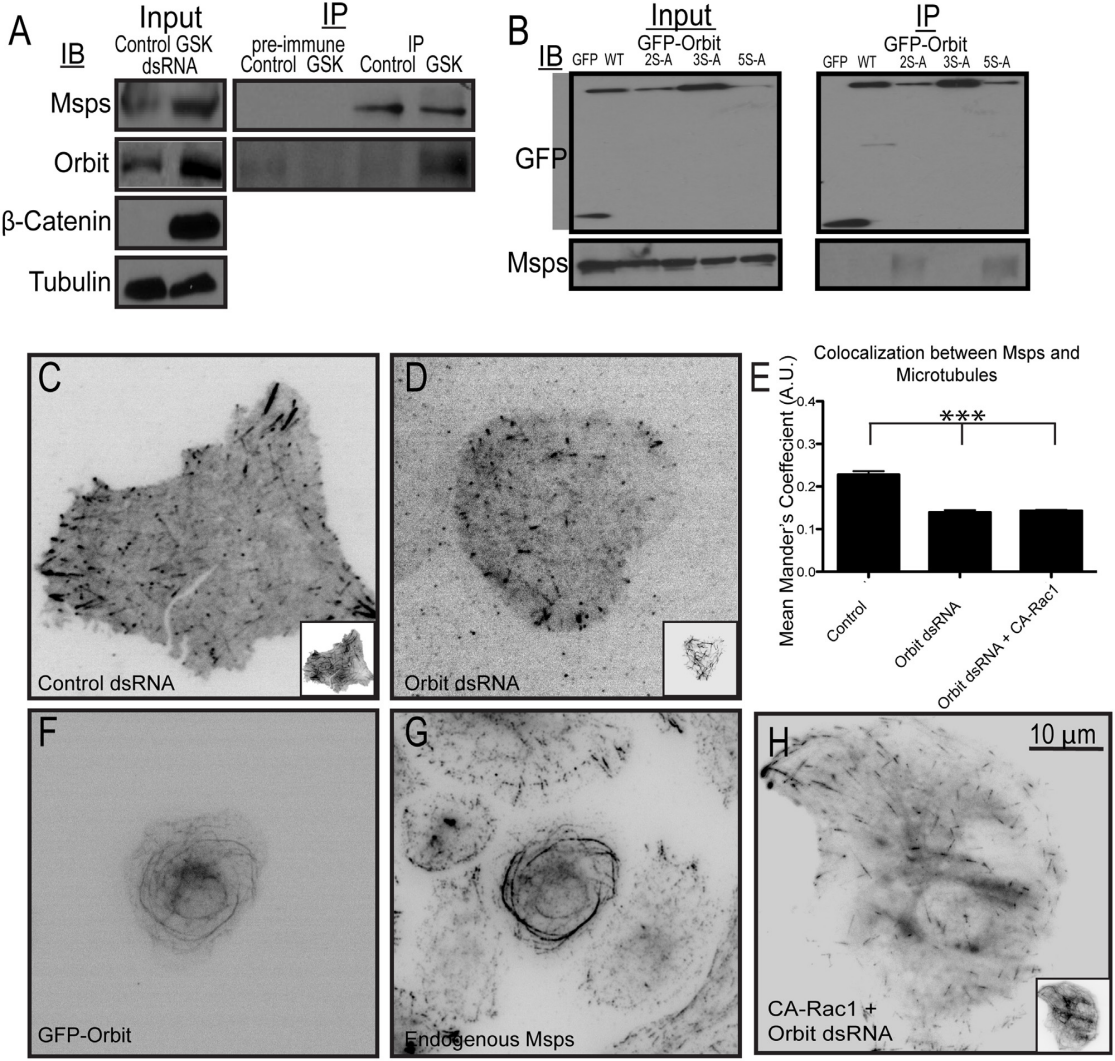
Orbit and GSK3β levels regulate Msps recruitment to the microtubule lattice. (A) Orbit coimmunoprecipitates with Msps from cells depleted of GSK3β using RNAi. GSK3β depletion was assessed using β-catenin levels, with tubulin as a loading control. (B) Msps coimmunoprecipitates with non-phosphorylatable mutants of Orbit. Immunoprecipitations were performed from cells depleted of endogenous Orbit using dsRNA targeting the 5'UTR of the gene and rescued with the indicated GFP-tagged Orbit constructs. (C-D) Msps-GFP was coexpressed with tRFP-α-tubulin in cells treated with control (C) or Orbit (D) dsRNA. Tubulin images are shown as insets. (E) Changes in co-localization of Msps were measured using the Mander’s coefficient, n = 90 cells from three experiments. *** p<0.0001 (F) GFP-Orbit was overexpressed in cells and stained for endogenous Msps (G). (H) Msps-GFP was coexpressed with a dual expression vector encoding tRFP-α-tubulin and CA-Rac1 in cells depleted of Orbit using RNAi.

In order to test whether the Orbit-Msps interaction is regulated by GSK3β, we depleted GSK3β using RNAi, immunoprecipitated Msps, and immunoblotted for Orbit. Immunoprecipitations were performed from S2 cells grown on tissue culture plastic, which do not spread under normal growth conditions, and exhibit basal Rac activity. In cells treated with control dsRNA, Orbit did not immunoprecipitate with Msps (Fig 3A), however, the two proteins did immunoprecipitate from GSK3β-depleted cells (Fig 3A). This result indicates that Orbit can interact with Msps when GSK3β is not active and when Orbit is likely not phosphorylated. In cells plated on concanavalin A to induce Rac-mediated cell spreading, we predict Orbit and Msps interact in the cell periphery where Msps is bound to the microtubule lattice. These results confirm a previous report that Orbit and Msps interact genetically and biochemically in *Drosophila* [35]. When we performed immunoprecipitations on cells lacking endogenous Orbit and expressing non-phosophorylatable Orbit mutants we found that the 2S->A and 5S->A mutants immunoprecipitate with Msps (Fig 3B). These data are consistent with the localization data, further indicating that when Orbit is phosphorylated it cannot interact with Msps.

To further test whether phosphorylation of Orbit regulates its ability to interact with Msps, we generated phosphomimetic mutations in Orbit in which the five serines were changed to aspartic acids (serines are shown in Fig 2A). To test whether these mutations affected Rac-GSK3β signaling on Orbit, we examined their localization in cells expressing CA-Rac1, which normally causes Orbit to bind along the lattice (Fig 1B). We also examined the effect of these mutations on endogenous Msps localization; if they disrupt the Msps-Orbit interaction, we predicted that they would prevent Msps from binding to the lattice as was seen with Orbit depletion (Fig 3D). All of these experiments were performed in cells depleted of endogenous Orbit by RNAi. As has been previously reported, the 3S->D had little effect on Orbit [8] or Msps localization. When expressed in control cells, the 3S->D mutation localized to microtubule plus ends, however, in cells expressing CA-Rac1 it localized to the microtubule lattice like the wild-type protein (S2Q, S2R and S2P Fig). Localization of Msps was also not affected and it remained on the lattice in the periphery (S2S and S2T Fig). Also consistent with Kumar et al. [8], the Orbit 2S->D mutation had a greater effect: it localized to plus ends when expressed in control cells but exhibited decreased localization to the microtubule lattice in cells expressing CA-Rac1 compared to wild type Orbit (S2M, S2N and S2P Fig). In addition Orbit 2S->D recruited endogenous Msps to the microtubule lattice to a lesser extent than wild type Orbit (S2O and S2T Fig). Mutation of all five serines, 5S->D had the largest affect. In control cells it localized to microtubule plus ends, however, we also observed an increase in the cytoplasmic pool. We speculate that this increased soluble pool is due to the fact that EB1 interaction SxIP motif is directly upstream of the phosphorylation sequence and addition of several aspartic acids in this region weakens the binding as had been previously described [8] (S2U Fig). When expressed with CA-Rac1, Orbit 5S->D remained on the plus end (S2V and S2P Fig). Expression of this mutant also prevented endogenous Msps from binding to the microtubule lattice (S2W and S2T Fig). When we performed immunoprecipitations from cells depleted of endogenous Orbit and rescued with these phosphomimetic mutants, we found that it could not coimmunoprecipitate with Msps (S2X Fig). These data are consistent with the model that when Orbit is phosphorylated it cannot interact Msps and that this interaction is required for Msps lattice binding.

We next asked if this protein-protein interaction regulated how Msps interacts with microtubules. First, we depleted Orbit from S2 cells and used immunofluorescence to localize Msps. In the absence of Orbit, Msps fails to localize to the microtubule lattice in the periphery and is instead targeted exclusively to microtubule plus ends (Fig 3C–3E, S1G, S1I and S1N Fig). Although a recent study implicated that mammalian Orbit is required to target EB1 to growing microtubule plus ends, we note that depletion of Orbit in S2 cells does not affect the localization of either EB1 or Sentin, which remain on the plus end [36] (S3A, S3B and S3E–S3G Fig). Next, we overexpressed Orbit in S2 cells to determine if this affected Msps localization. Overexpressed Orbit binds along the full lengths of microtubules leading to their hyperstabilization and bundling in the central region of the cell [37] (Fig 3F). We observed that ectopically overexpressed Orbit recruits endogenous Msps to microtubule bundles (Fig 3G), indicating that Orbit is sufficient to recruit Msps to the microtubule lattice. To test if this interaction is important for Msps regulation by Rac, we performed RNAi to deplete Orbit, transfected the cells with CA-Rac1, and plated them on concanavalin A for immunofluorescence. In the absence of Orbit, Msps can no long respond to CA-Rac1 by binding the microtubule lattice throughout the cell (for comparison see Figs 1D and 3H). Based on these data, we conclude that Msps and Orbit interact in cells and this protein-protein interaction is regulated by GSK3β. When GSK3β is inactive and Orbit is not phosphorylated, it can interact with Msps, allowing Msps to bind to the lattice. When GSK3β is active, in the cell cortex, it phosphorylates Orbit and prevents this interaction.

### Sentin Is Necessary for Msps and Orbit to Interact

**Fig 4:**
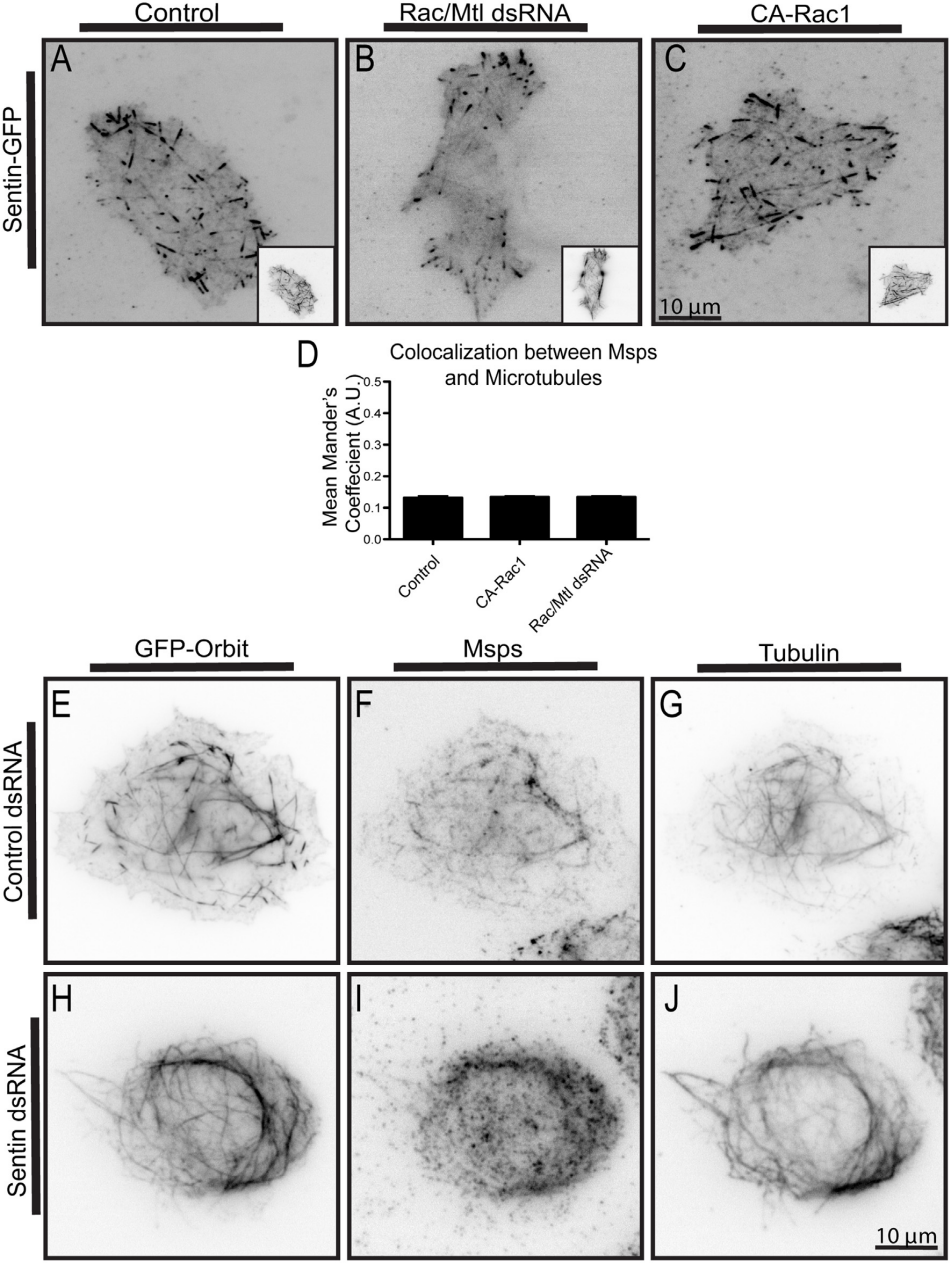
Sentin expression is required for the Orbit-Msps interaction, but its localization to microtubules is not regulated by Rac. (A-C) Sentin-GFP was co-expressed with tRFP-α-tubulin in control cells (A), in cells depleted of Rac1/Rac2/Mtl using RNAi (B), or together with CA-Rac1 (C) Tubulin images are shown as insets. (D) Changes in co-localization of Msps were measured using the Mander’s coefficient, n = 90 from three experiments. (E-J) The localization of overexpressed GFP-Orbit, endogenous Msps, and α-tubulin is shown for cells treated with control (E-G) or Sentin (H-J) dsRNAs.

In order to localize to the growing plus end of the microtubule, many +TIPs bind the scaffolding protein EB1 through a SXIP motif [38]. Msps, however, interacts with EB1 indirectly through a bridging protein, Sentin, in order to localize to plus ends [27]. We found that Sentin immunoprecipitates with Orbit in S2 cells in both control and GSK3β depleted cells (S3H Fig). Sentin's dynamics, along with the fact that it recruits Orbit and Msps to the plus end, suggest it is a functional homolog of mammalian SLAIN1/2 [39]. Localization of Sentin and EB1 are not affected by expression of CA-Rac1 or by Rac1/Rac2/Mtl RNAi, indicating that these two proteins are upstream of Rac signaling in this pathway (Fig 4A–4D and S3C, S3D and S3G Fig). To test the hypothesis that Sentin acts as a bridge between Msps and Orbit, we overexpressed Orbit and examined the dependencies for recruitment of Sentin or Msps to microtubule bundles. We found that, similar to Msps, Sentin is also recruited to the microtubule lattice upon overexpression of Orbit (S3I and S3J Fig). When Sentin was knocked-down, Orbit overexpression failed to recruit Msps to the microtubule lattice (Fig 4E–4J). However, when Msps was depleted, Sentin could still be recruited to microtubules (S3K and S3L Fig). These data indicate that Sentin is necessary for Orbit and Msps to interact, but that it can interact with both proteins individually. We propose that in the cell cortex, where Rac activity is low and GSK3β activity is high, Sentin binds to both proteins at the plus end. In the periphery of the cell where Rac is active, Orbit is not phosphorylated by GSK3β and Sentin can act to bring Msps and Orbit together in order to facilitate their interaction.

### The C-Termini of Msps and Orbit Interact

**Fig 5:**
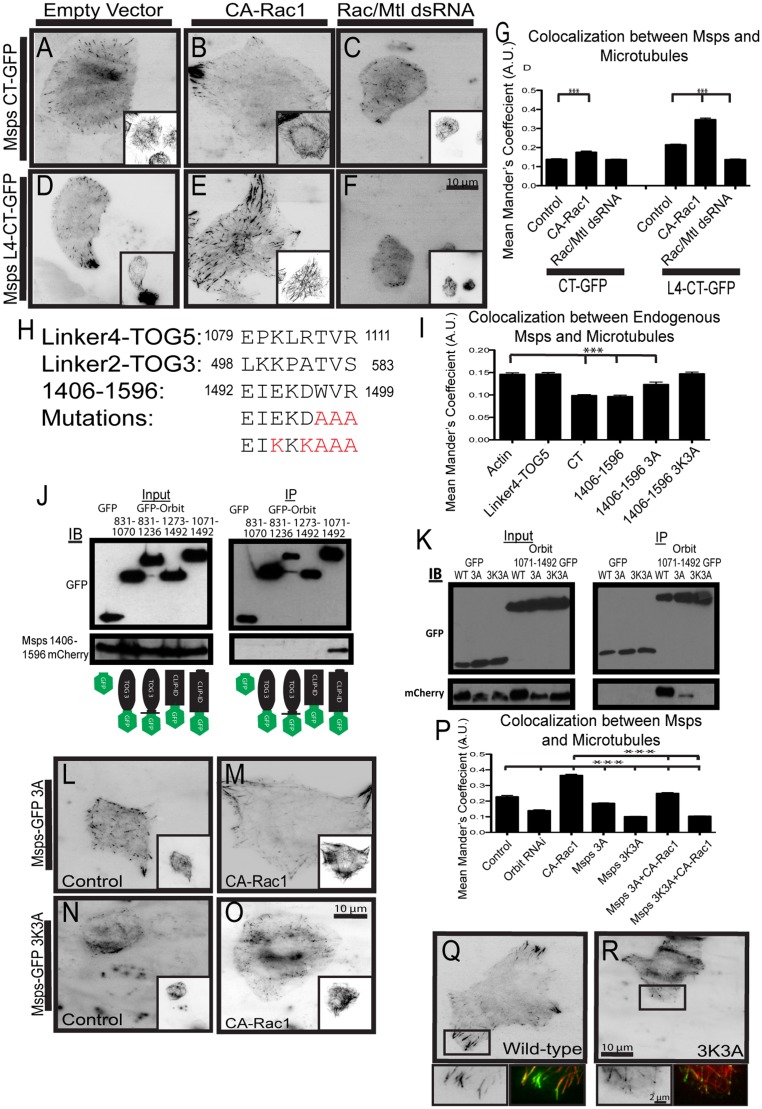
Msps 1406–1506 interacts with Orbit. (A-C) MspsCT-GFP was expressed in cells with a dual expression vector containing tRFP-α-tubulin alone (A) or with CA-Rac1 (B) and also in cells with Rac1/Rac2/Mtl depletion (C). (D-F) Msps L4-CT-GFP was expressed in cells with a dual expression vector containing α-tubulin-tRFP alone (D) or with CA-Rac1 (E) and also in cells with Rac1/Rac2/Mtl depletion (F). Tubulin images are shown as insets. (G) Changes in co-localization of Msps were measured using the Mander’s coefficient, n = 90 cells from three experiments. *** p<0.0001 (H) Alignment of sequences contained in Msps Linker4-TOG5, Linker2-TOG3 and 1406–1596, residue number is indicated to the right and left of the residue. (I) Changes in co-localization of endogenous Msps and microtubules due to competition with Msps C-terminal and mutant fragments, n = 90 cells from two experiments. *** p<0.0001 (J) mCherry-tagged Msps 1406–1596 coimmunoprecipitates with C-terminal Orbit residues 1071–1492. (K) mCherry-tagged Msps 1406–1596 mutants fail to coimmunoprecipitate with C-terminal Orbit residues 1071–1492. (L-M) Msps-GFP 3A was expressed in cells with a dual expression vector containing tRFP-α-tubulin alone (L) or with CA-Rac1 (M). (N-O) Msps-GFP 3K3A was expressed in cells with a dual expression vector containing tRFP-α-tubulin alone (N) or with CA-Rac1 (O). Tubulin images are shown as insets. (P) Co-localization between Msps and microtubules were measured using the Mander’s coefficient, n = 90 cells from three experiments. *** p<0.0001 (Q) Localization of wild-type Msps, insets represented with a rectangle highlighting the periphery. (R) Localization of Msps 3K3A, insets represented with a rectangle highlighting the periphery.

We next wanted to identify the domain of Msps that interacts with Orbit. We focused on the C-terminus of Msps as it is required for proper localization within the cell [21]. We previously identified a microtubule lattice-binding site in Msps composed of the inter-TOG linker 4 region (L4) and the fifth TOG domain (residues 1079–1405). This site was both necessary and sufficient to recruit full-length Msps to the microtubule lattice and localized to microtubules when expressed in isolation [21]. In contrast, the C-terminal region of Msps (MspsCT, residues 1406–2050) localizes to growing microtubule plus ends [21] (Fig 5A). A construct composed of both regions, Linker4 to the C-terminus (L4-CT, residues 1079–2050) exhibits localization patterns that mimic full-length Msps (Fig 5D). To test if Rac1 could alter the localization of these fragments, we either co-expressed them with CA-Rac1 or transfected them into cells depleted of Rac1/Rac2/Mtl using RNAi. Both L4-CT and the MspsCT responded to changes in Rac activity. L4-CT responded to CA-Rac1 by binding to microtubules throughout the cell and lost its peripheral lattice association when Rac1/Rac2/Mtl were knocked down (Fig 5D–5G). MspsCT localizes to growing microtubule plus ends in control cells, but when CA-Rac1 is expressed, it localized to the microtubule lattice in the cell periphery, and closely resembled full-length Msps localization (Fig 5A, 5B and 5G). Rac1/Rac2/Mtl knock down has no effect on MspsCT localization to plus ends (Fig 5C and 5G). These data indicate that Msps has two distinct microtubule-binding activities that are responsive to Rac activation. We decided to focus on the MspsCT fragment since it was the smallest piece that responded to Rac1.

In order to narrow down the region of Msps that binds Orbit, we developed a cell-based colocalization assay. In our assay we overexpressed fragments of the Msps C-terminus and probed for endogenous Msps lattice binding in the cell periphery; if the fragment being overexpressed bound to Orbit it should outcompete endogenous Msps for binding, thus preventing endogenous Msps from binding the lattice. As a negative control we transfected cells with actin; in these cells endogenous Msps displayed a normal localization (quantified Fig 5I). We designed the C-terminal expression constructs based on secondary structure prediction (S3M Fig). We found that the smallest piece that could out-compete endogenous Msps for Orbit binding was residues 1406–1596 (Fig 5I, S3M Fig). In cells expressing Msps 1406–1596, endogenous Msps localized to the plus end, but did not bind to the lattice, indicating that the 1406–1596 fragment competed for binding to Orbit.

We examined the level of sequence identity in Msps 1406–1596 with homologs from other higher eukaryotes and identified a motif (EIEKDWVR) that was conserved across taxa. We note that this sequence exhibits similarity to microtubule binding motifs in the inter-TOG linker regions 2 and 4 (residues 498–583 and 1079–1181) [21] (Fig 5H; S3N Fig underlined residues). We speculated this motif mediated a protein-protein interaction with Orbit; to test this possibility we used alanine and charge-reversal mutagenesis to ablate the motif and asked if the mutants could still compete with endogenous Msps for binding to Orbit in cells (Fig 5H). In cells expressing Msps 1406–1596 with WVR mutated to AAA (3A), endogenous Msps could bind to the lattice in the periphery but to a lesser extent, indicating the 3A mutation could still weakly bind and outcompete endogenous Msps for endogenous Orbit. In cells expressing Msps 1406–1596 with EKDWVR mutated to KKKAAA (3K3A), endogenous Msps exhibited normal lattice association in the periphery, indicating that the 3K3A mutant could not bind and outcompete endogenous Msps for endogenous Orbit (Fig 5I). These data suggested that the Orbit binding site within Msps is within the C-terminus and encompasses residues Msps1406-1596.

In order to identify the region of Orbit that binds to Msps we overexpressed fragments of Orbit that are able to bind and bundle microtubules similar to full length Orbit (S4A and S4B Fig; Fig 3F). We then stained for endogenous Msps to see which fragments could recruit endogenous Msps to the lattice indicating it is able to bind Msps (S4B and S4C Fig; Fig 3F and 3G). We found that a construct containing the third TOG domain to the C-terminus of Orbit (TOG3-CT, residues 831–1492; S4C Fig) was the only fragment that could recruit endogenous Msps to the microtubule lattice (S4C Fig). To narrow down the Msps binding site, we performed immunoprecipitations with Msps 1406–1596 and different pieces of the Orbit TOG-CT fragment (Fig 5J; fragments tagged with GFP in cartoon at bottom). We found that Orbit residues 1071–1492, a region embodying the linker region and CLIP interacting domain, was able to pull down with Msps 1406–1596 (Fig 5J). Msps1406-1596 3A exhibited decreased interaction with Orbit 1071-1492-GFP and Msps1406-1596 3K3A failed to immunoprecipitate at all (Fig 5K). Thus, our results indicate that Orbit interacts with Msps through its C-terminus including the CLIP-binding domain.

In order to determine how the Orbit-Msps interaction contributed to Msps localization, we next made the 3A and 3K3A mutations in full-length Msps-GFP. The 3A mutation had a modest effect on Msps lattice binding and, while it still responded to CA-Rac1 co-expression, it did so to a slightly lesser extent. (Fig 5L, 5M and 5P). The larger 3K3A mutation completely prevented localization of Msps to the microtubule lattice (Fig 5N). This mutation also had a dramatic effect on Msps tip localization, causing Msps-GFP to adopt a more punctate localization at the extreme distal microtubule tip (Fig 5N and 5R). Msps(3K3A)-GFP failed to respond to CA-Rac1 expression and retained its punctate morphology at the microtubule plus end (Fig 5O and 5P). This result is reminiscent of our previous work that showed mutations of the lattice binding regions of Msps (residues 498–583 and 1099–1111) exhibit similar punctate localization patterns [21]. In summary, these data indicate that Orbit and Msps can directly interact through their C-termini and that this interaction is required for proper localization of Msps to the microtubule lattice in the cell periphery and also for proper plus end association.

### The Msps-Orbit Interaction Decreases Microtubule Growth Rates in the Cell Periphery

**Fig 6:**
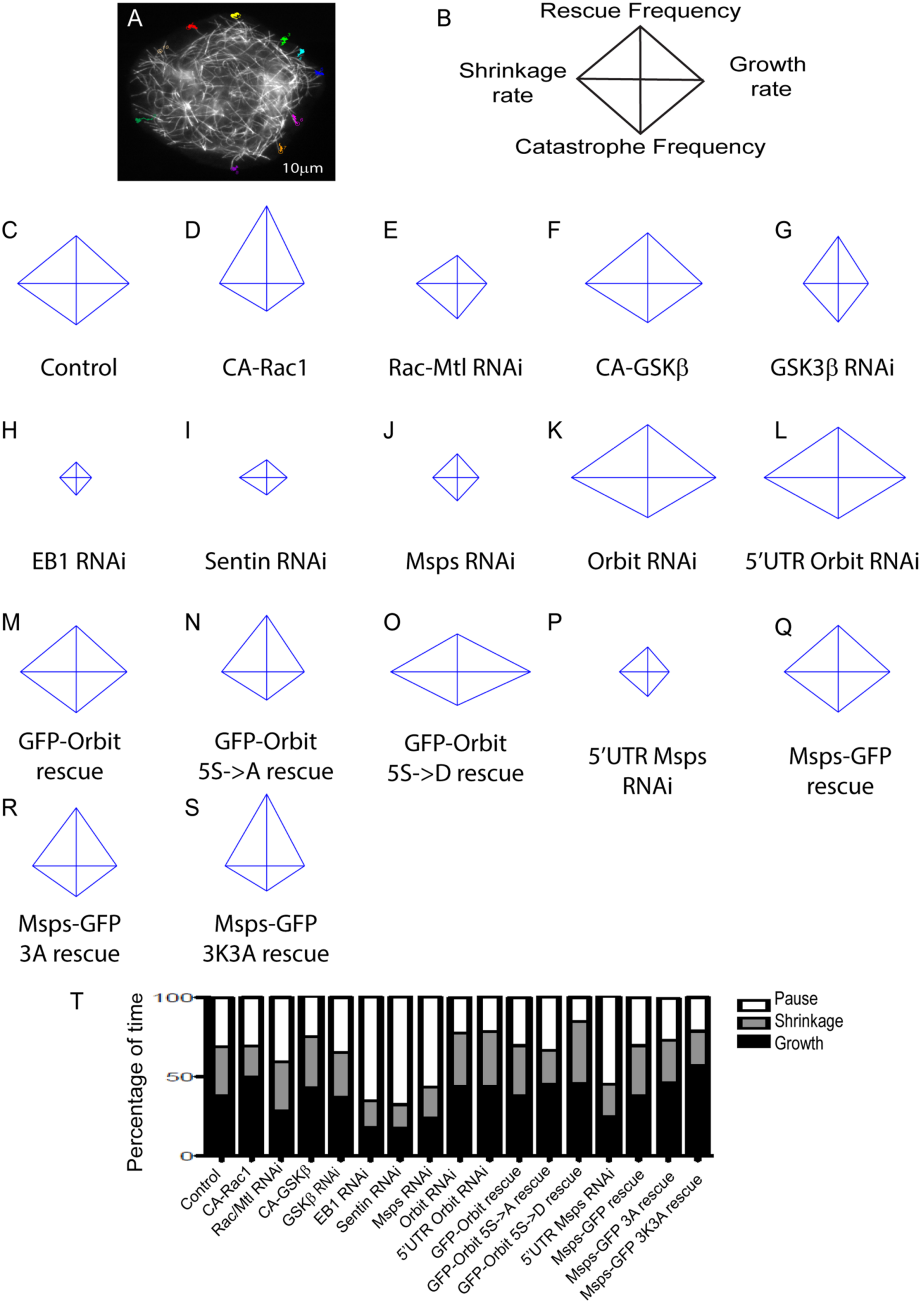
Measurement of microtubule dynamics. (A) Representative image of a cell with microtubules that were tracked. Each number represents a separate track. 10 microtubules per cell were tracked at the periphery of the cell for at least 30 seconds. 30 cells per condition were tracked, the experiment was repeated three times, with 10 cells per replicate. Between 5–10 microtubules were tracked per cell. (B) Representative schematic of a diamond plot with the rates on the x-axis, growth (right) and shrinkage (left) and frequency on the y-axis, rescue (up) and catastrophe (down). (C-R) Diamond graphs of microtubule dynamics under each condition. Diamond graphs are jointly normalized. (S) Graphs showing percentage of time microtubules spent in growth, shrinkage and pause with each condition.

In order to ascertain the function of the Msps-Orbit interaction on microtubule dynamics, we established a system to measure parameters of microtubule dynamic instability in S2 cells. We expressed TagRFP-α-tubulin under control of the Tub84 promoter and manually tracked microtubules at the cell periphery where dynamics of individual microtubules are easily delineated over time (Fig 6A; S1 Table). We present the results of these experiments using four-axis diamond graphs; these plots show the rates on the horizontal axis (left: shrinkage; right: growth) and frequencies on the vertical axis (down: catastrophe; up: rescue) [34] (Fig 6B). Changes in the shape of the graphs indicate changes in the microtubule dynamics with the condition. We first wanted to determine how the different regulatory molecules in this pathway influence microtubule dynamics. Expression of CA-Rac1 caused more persistent microtubule growth as compared to control cells (Fig 6C and 6T), with slight decreases in the rates of growth and shrinkage and more time spent in growth (Fig 6D and 6T; S1 Table). We note that the slower growth rate may be attributable to the increased actin retrograde flow in these cells as has been previously described in mammalian cells [2] (S4D Fig). Conversely, RNAi to deplete Rac1/Rac2/Mtl caused microtubule dynamics to decrease and they spent a greater fraction of their lifetime in a paused state [2] (Fig 6E and 6T; S1 Table). Expression of CA-GSK3β caused an increase in the amount of time microtubules spent in growth and a corresponding decrease in microtubule pause, similar to results described in mammalian cells [8] (Fig 6F, 6S and 6T; S1 Table). RNAi of GSK3β resulted in decreased cell viability and a decrease in microtubule dynamics (Fig 6G and 6T; S1 Table). We also examined the phenotype caused by depletion of the +TIPs in this pathway—EB1, Sentin, Msps and Orbit—and confirmed previously published effects on microtubule dynamics. EB1, Sentin and Msps have been shown to promote microtubule dynamics, and depletion of any of these three protein results in microtubules that spend most of their time in pause [20,21,27,28] (Fig 6H–6J and 6T; S1 Table). Orbit knock down, however, increases microtubule dynamics, consistent with its microtubule stabilizing activity [37] (Fig 6K and 6T; S1 Table).

We next measured microtubule dynamics in S2 cells depleted of endogenous Orbit or Msps and rescued with the mutants that decrease their interaction. For the rescue experiments, we performed RNAi against Msps or Orbit with dsRNAs targeting the 5’UTR of each gene. Depletion with these dsRNAs had a similar effect on microtubule dynamics as dsRNAs targeting the coding sequence; when Msps-GFP or GFP-Orbit where transfected to rescue the depletion, microtubule dynamics were rescued to control levels (Fig 6C, 6J–6M, 6O–6Q and 6T; S1 Table). Since GSK3β knock down was toxic to cells, we decided to rescue Orbit knock down with the 5S->A Orbit mutation, to specifically test the role of GSK3β-mediated phosphorylation of Orbit on this pathway. When we rescued with this mutant, microtubules spent more time in both growth and pause, and less time in shrinkage (Fig 6N and 6T; S1 Table). When Orbit depletion was rescued with the phosphomimetic 5S->D mutation, microtubules spent more time in growth and shrinkage than in pause and had decreased rescue, stall and pause frequencies (Fig 6O and 6T). When Msps knock down was rescued with the 3A mutation, there was a modest effect on microtubule dynamics, consistent with the fact that Msps lattice association was only modestly affected (Fig 6R and 6T; S1 Table). However, the Msps 3K3A mutation exhibited a more significant effect, consistent with its dramatic effect on Msps localization. These mutations increased the time spent in growth and decreased time spent in pause and shrinkage, and microtubules grew continuously at a slower velocity (Fig 6S and 6T; S1 Table). In summary, disruption of any member of this pathway had a pronounced effect on microtubule dynamics at the cell periphery. Mutations that disrupt this pathway demonstrate that it is important for regulating microtubule growth and pause: non-phosphorylatable Orbit causes an increase in growth and pause, while mutations that prevent the Msps-Orbit interaction lead to an increase in growth.

## Discussion

Subcellular regulation of microtubule dynamics is important during the establishment of cell polarity, however, the molecular mechanisms underlying the regional control of dynamic instability remain ill defined. Work in mammalian cells has demonstrated that the small GTPase Rac promotes persistent microtubule growth through three distinct mechanisms. The first is through the GSK3β-CLASP pathway in which Rac locally inhibits GSK3β in the lamellae causing CLASP to localize to the microtubule lattice and suppress their dynamics [7]. The second mechanism is through Rac-activated PAK kinases, which then phosphorylate and inactivate the microtubule destabilizing protein stathmin [4]. Stathmin functions as a tubulin sequestering protein and promotes microtubule depolymerization; its local inhibition at the leading edge decreases the probability of microtubule catastrophe. The third mechanism is through cortical capture of microtubules at the leading edge through an interaction between the +TIP CLIP-170 and the Rac effector IQGAP1. Although all of these mechanisms contribute to localized regulation of dynamic instability, they do not explain how Rac activation induces persistent microtubule growth.

In the present study, we have identified the XMAP215 homolog, Msps, as a downstream effector of the Rac pathway and describe a novel regulatory mechanism for Msps through a protein-protein interaction with the microtubule stabilizer Orbit and the scaffolding protein Sentin. In S2 cells, the *Drosophila* CLASP homolog Orbit localizes to microtubule plus ends, but binds to the microtubule lattice upon expression of active Rac1 or depletion of GSK3β. These observations are similar to the dynamics of CLASP in mammalian cells and suggest that this mode of regulation is conserved. Activation of Rac or depletion of GSK3β promotes Msps binding to the microtubule lattice and this localization requires Orbit. Our data suggests that, like CLASP, Orbit is directly phosphorylated by GSK3β which prevents it from interacting with and recruiting Msps to the microtubule lattice. The Orbit-Msps interaction further requires another +TIP, Sentin. As the localization of Sentin at the microtubule plus ends is not regulated by Rac-GSK3β, it is likely serving a scaffolding function to promote interactions between Msps and Orbit. We mapped the protein-protein interaction sites on Msps and Orbit to their C-termini and found that mutations that block their interaction severely perturb microtubule dynamics. Both a non-phosophorylatable Orbit mutant and a mutant that prevented the Msps-Orbit interaction lead to more persistent growth, with the non-phosphorylatable Orbit mutant also causing an increase in microtubule pause. This may indicate that this interaction is important for persistent microtubule growth downstream of Rac-GSK3β.

A growing body of evidence indicates that +TIPs exhibit cross-talk with one another to regulate microtubule dynamics in response to upstream regulatory cues. Our results indicate that Msps and Orbit function together during interphase to regulate dynamic instability in response to Rac and GSK3β activity. It is well established that members of the XMAP215 family promote microtubule dynamics by catalyzing microtubule polymerization and depolymerization. These activities are conserved in *Drosophila* Msps; microtubules in S2 cells lacking Msps are less dynamic, spending most of their lifetime in a pause state. In contrast, Orbit acts to suppress microtubule dynamics and promotes their stability. Thus, Msps and Orbit would be seem to regulate microtubule dynamics antagonistically, a functional relationship supported by recent genetic studies [35]. However, our results indicate that the two proteins share a more complex interaction.

Msps exhibits two distinct localization patterns on microtubules in S2 cells- at the plus ends of microtubules and along the distal microtubule lattice in the periphery. Our data support the model that these different modes of microtubule association represent functionally distinct pools of Msps. First, we identified the Msps-Orbit interaction sites, mutated them to ablate the interaction, and used these mutants to rescue cells depleted of either endogenous Msps or Orbit. Expression of either Msps-GFP 3A or 3K3A was able to rescue microtubule dynamics as compared to Msps-depleted cells. However, microtubules in these cells exhibited abnormally high frequencies of rescue and low frequencies of catastrophe, spending more time in growth and less in shrinkage compared to control cells. Cells expressing GFP-Orbit with the GSK3β phospho-acceptor sites mutated to alanine (5S->A) suppressed Orbit RNAi-induced increases in dynamic instability, but microtubules in these cells also exhibited higher frequencies of rescue, lower frequencies of catastrophe, and more time in the pause state compared to control cells. These results indicate that Msps must interact with Orbit in order to properly regulate microtubule dynamics in the cell periphery. Second, when we examined the growth rates of microtubules by tracking EB1-GFP, we noted that EB1 comets in the cell periphery exhibit slower velocities than those in the cell cortex. These differences likely reflect interactions between growing microtubules and lamellipodial actin undergoing retrograde centripetal flow in the cell periphery [2]. Recent work has also shown that EB1 comets are structurally different in the cortex versus the periphery, so the differences may be explained by changes in the microtubule as well [40]. However, when we compared EB1 comets on microtubules with Msps at the plus end to those that had Msps localized along the distal lattice, we discovered that the latter exhibited a significantly slower rate of growth. Collectively, our results indicate that the Msps-Orbit interaction "tunes" microtubule dynamics in response to Rac activation in the cell periphery. We suggest that Msps could be shunted onto the lattice to act as a localized “sink” that attenuates its activity as a microtubule polymerase. This inactive pool may serve as a mechanism to partially suppress Msps activity so that microtubules grow at specific rates upon reaching the edge of the cell. The mechanism of how Msps regionally governs microtubule dynamics presents an intriguing problem; future studies employing biochemical reconstitution of microtubule dynamics with recombinant +TIPs and their regulators will likely be required to address these models.

One puzzling aspect of our study is that, despite the biochemical and functional evidence for the Msps-Orbit interaction, we were never able to detect colocalization of Msps and Orbit on the microtubule lattice in the cell periphery under unperturbed conditions. We speculate that this protein-protein interaction is transient, occurring at the plus end, but is required for some conformational change in Msps that unmasks its microtubule lattice-binding activity. We previously identified two lattice-binding sites in the inter-TOG linker regions that seem to be inactive while the protein is localized to plus ends [21]. Widlund et al. also confirmed these results using in vitro reconstitution assays [41]. It is possible, however, that Orbit does localize to the lattice in the cell periphery, but at levels so low we were unable to detect in living cells using the probes at our disposal. A third possibility is that Orbit is able to alter the structure of the microtubule lattice proximal to the plus end in order to promote lattice binding of Msps. This alteration could represent a change in the local nucleotide state of the polymer as a recent study indicated that mammalian CLASPs are able to promote GTP hydrolysis by polymerized tubulin [36]. Alternatively, Zhang et al. recently showed that EB1 family members promote structural transitions within the microtubule lattice that favor GTP hydrolysis and compaction of the lattice itself. Perhaps similar localized changes in microtubule structure signal to Msps to transition from tip-association to lattice binding [42]. Further work will be required to understand how these proteins interact to regulate their respective functions and we expect that in vitro reconstitution assays will prove valuable to advance our understanding of this protein-protein interaction.

It is interesting to note that the localization patterns for Msps and Orbit we observe in *Drosophila* cells seem to exhibit the converse relationships to those described for XMAP215/CH-TOG and CLASP in mammalian cells [7,43]. Msps also differs from XMAP215 and ch-TOG through its lack of ability to either bind directly to EB1 or independently recognize growing microtubule ends. It is possible that the interaction with Orbit developed to increase Msps’ ability to target the microtubule plus end. This interaction may also be present in mammalian cells, where it may serve to modulate growth rates of microtubules. Although Msps and XMAP215/CH-TOG exhibit high degrees of identity overall [10], it will be interesting to compare their relative activities in living cells using Msps to replace CH-TOG, and vice-versa, using heterologous systems.

Our data point to an outstanding question about how this localized regulation of dynamic instability impact behavior at the level of the cell. Dynamic microtubules exhibit a complex, bidirectional cross-talk with the Rho family of small G proteins [44]. We suggest that Msps and other XMAP215 family members are critical components of these pathways. In migrating cells, for example, Rac activity promotes processive microtubule growth while microtubule dynamics also promote Rac activation [1]. We predict that Msps/XMAP215 family members are likely to participate in this positive feedback look and are, therefore, likely to play crucial roles in cell motility. Microtubules are also essential for directed membrane traffic to the leading edge (reviewed in Rodriguez et al, 2003). Msps-induced microtubule growth may also contribute to this polarized delivery of cargo to the front of motile cells. In order to address these fascinating questions, the Msps-Orbit interaction will have to be addressed in the context of migratory cell lines or, better still, within the developing embryo.

**Fig 7:**
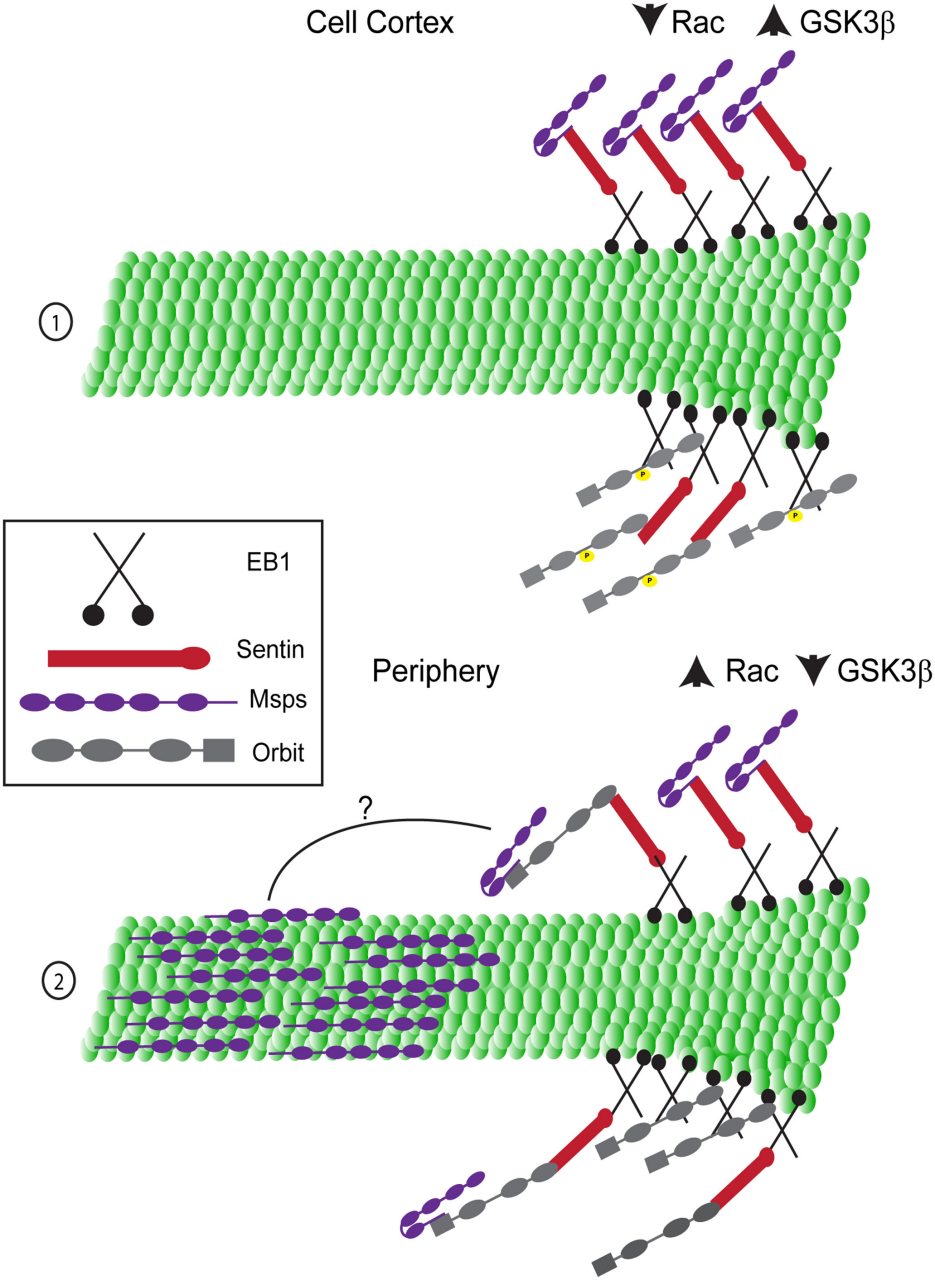
Model. The interaction between Msps and Orbit is required for Msps lattice association. (1) At the plus end of a growing microtubule (green) in the cortex of the cell, Msps (purple) can localize to the plus end through its interaction with Sentin (red), which can bind EB1 (black). Msps cannot interact with the lattice and we speculate it is in a folded conformation in which its lattice binding domains are masked. Orbit (grey) is phosphorylated by GSK3β and can interact with the plus end by binding either EB1 directly or Sentin. (2) At the plus end of the microtubule in the periphery, both Msps and Orbit bind to the plus end through the same associations as in the cell cortex. Orbit is no longer phosphorylated by GSK3β and can interact with Msps. The two proteins interact through their C-termini and this allows Msps to bind to the lattice further back from the plus end, possibly by changing the conformation of Msps to expose its lattice binding domains.

In our model (Fig 7) in the cortex of the cell (1), Rac activity is low and therefore GSK3β is active, leading to phosphorylation of Orbit on 5 serine residues. Both Orbit and Msps are at the plus end, but cannot interact with each other. Msps is localized to the plus end through its interaction with Sentin. Orbit can bind either Sentin or EB1 to target the plus end. In the periphery of an S2 cell (or the leading edge of a migrating cell (2)), Rac is active, which leads to the local inactivation of GSK3β and dephosphorylation of Orbit. Orbit is still on the plus end, but is now able to interact with Msps, allowing Msps to bind to the lattice. How this interaction allows Msps to bind the lattice with Orbit remaining on the plus end remains to be determined. We hypothesize that when Msps is at the plus end it is in a closed conformation where the C-terminus covers the Linker4-TOG5 region that can bind the microtubule lattice (Msps (purple) (1)). When Msps and Orbit bind to one another, this causes Msps to adopt an open conformation, exposing the lattice binding region which allows Msps to diffuse along to lattice (Msps (purple) (2)).

## Supporting Information

S1 Fig Endogenous Orbit and Msps are regulated by Rac-GSK3β signaling. (A) EB1 and Msps expressing cells, merge image shows Msps in green and EB1 in red. The different colored boxes represent the different regions of the cells in which EB1 comets were tracked, purple represents the cortex of the cell, green represents the periphery and blue represents the lattice bound population. (B) Graph of the EB1 velocities in each population, colors correspond to the region of the cell. 20 cells were imaged and 5 comets per location in the cell were tracked results are from three experiments *** p<0.0001 (C) Control cell expressing a dual expression vector with tRFP- α-tubulin alone. (D) Cell expressing a dual expression vector with tRFP- α-tubulin and CA-Rac in the second site. (E) Endogenous Orbit and α-tubulin were stained in control cells and cells expressing CA-Rac1 (F). CA-Rac expressing cells were identified as those that were able to spread on glass coverslips without ConA. Rac1/Rac2/Mtl (J) or GSK3β (K) were knocked down with dsRNA and the cells stained for endogenous Orbit and α-tubulin. (G) Endogenous Msps and α-tubulin were stained in control cells (G) and cells expressing CA-Rac1 (H). Orbit (I), Rac1/Rac2/Mtl (L), or GSK3β (M) were knocked down with dsRNA in cell stained for endogenous Msps and α-tubulin. Tubulin images are shown as insets. (N-O) Changes in the co-localization of Msps (P) and Orbit (Q) with microtubules were measured using the Mander’s coefficient, n = 90 cells from three experiments. An increase indicates increased lattice binding and a decrease indicates decreased lattice binding. *** p<0.0001 (P-S) Msps-GFP is expressed in cells with a dual expression containing tRFP-α-tubulin and CA-Cdc42 (P) or CA-Rho (R). Cdc42 (Q) or Rho (S) were knocked down with dsRNA in cells expressing tRFP-α-tubulin. Tubulin images are shown as insets. (T) Changes in the co-localization of Msps and tubulin was measured using the Mander’s coefficient, n = 90 cells from three experiments. (U) Levels of depletion were measured using a functional assay for cell spreading. For Rac1/Rac2/Mtl depletion, cell edges were scored for smooth or rough edges. Rough edges are characteristic of Rac depletion (Rogers et al., 2003). Successful depletion of +TIPs was measured by scoring the mitotic index using pH3 antibody.
(TIF)

S2 Fig Orbit is phosphorylated by GSK3β in the linker region between TOG2 and TOG3. (A-J) Controls to test the efficiency of the Mander’s coefficient. Actin was used as a negative control. (A) GFP Actin expressing cell before processing and after subtraction of the background and despeckling (B). (C) tRFP- α-tubulin expressing cell before (C) and after processing (D). Merged image of post processed cell shows Actin in green and Msps in red. (F) MAP2C GFP expressing cell pre (F) and post (G) processing. tRFP- α-tubulin expressing cell pre (H) and post (I) processing, (J) Merged image shows MAP2C in green and tubulin in red. (K) Graph of the Mander’s coefficient of the two controls, N = 90 cells from three experiments. (L) Graph of the number of objects per cell (EB1 comets) versus the Mander’s coefficient of that cell. Images from both control (black dots) and CA-Rac1 expressing cells (white dots) were used. (M-O) GFP-Orbit 2S->D was expressed in cells with a dual expression vector containing tRFP-α-tubulin alone (M) or with CA-Rac1 (N). (O) Endogenous Msps and α-tubulin were stained in cells transfected with 2S->D. (Q-S) GFP-Orbit 3S->D is expressed in cells with a dual expression vector containing α-tubulin-tRFP alone (Q) or with CA-Rac1 (R). (S) Endogenous Msps and α-tubulin were stained in cells transfected with 2S->D. (U-W) GFP-Orbit 5S->D was expressed in cells with a dual expression vector containing tRFP-α-tubulin alone (U) or with CA-Rac1 (V). (W) Endogenous Msps and α-tubulin were stained in cells transfected with 5S->D. Tubulin images are shown as insets. (P and T) Changes in co-localization of Orbit (P) and endogenous Msps (T) were measured using the Mander’s coefficient, n = 90 cells from two (endogenous Msps) or three (GFP-Orbit) experiments. *** p<0.0001. (X) Msps cannot coimmunoprecipitate with phosphomemetic mutants of Orbit. Immunoprecipitations were performed from cells depleted of endogenous Orbit using dsRNA targeting the 5'UTR of the gene and rescued with the indicated GFP-tagged Orbit constructs.
(TIF)

S3 Fig EB1 and Sentin localization is not regulated by Rac or Orbit. (A-D) EB1-GFP was expressed in cells with a dual expression vector containing tRFP-α-tubulin alone (A) or CA-Rac1 (D) and also in cells with Orbit (B) or Rac1/Rac2/Mtl depletion (C). (E-F) Sentin-GFP was expressed in cells with tRFP- α-tubulin with control (E) or Orbit depletion (F) Tubulin images are shown as insets. (G) Changes in co-localization of EB1 and Sentin were measured using the Mander’s coefficient, n = 90 cells from three experiments. (H) Immunoprecipitation of Sentin for Orbit. Pre-immune serum was taken from rabbits prior to injection with the Orbit antigen. GSK3β depletion was assessed using β-catenin levels, with tubulin as a loading control. (I-L) GFP-Orbit was overexpressed in cells stained for endogenous Sentin and α-tubulin with control (I-J) or Msps (K-L) depletion. (M) Schematic of the C-terminus of Msps colored by regions of predicted secondary structure. The extent of Msps binding to the microtubule lattice in cells expressing different Msps C-terminal constructs. Different constructs were scored as lattice binding, weak lattice binding, or no lattice binding. (N) CLUSTAL alignment of the Msps C-terminus with higher eukaryote homologs, *Xenopus laevis* XMAP215 and *Homo sapiens* ch-TOG. The underline denotes a region of sequence conservation.
(TIF)

S4 Fig Determining the Msps binding site in Orbit. (A) A schematic of the domain structure of Orbit and different fragments expressed in cells. (B) Examples of Strong, Weak and No colocalization of Msps with overexpressed Orbit. (C) Msps lattice association in cells expressing the different constructs was scored as strong, weak or no colocalization with lattice bound Orbit. (D) Rates of actin retrograde flow is altered only by changes in Rac activity. Actin retrograde flow was measured using kymographs of actin particle movement from the cell periphery to the cortex, n = 20 cells from three experiments. *** p<0.0001.
(TIF)

S1 Movie Microtubule dynamics in a spread S2 cell. Microtubules in the cortex of the cell have different dynamics from those at the periphery. S2 cell transfected with mCherry-tubulin. Images were acquired using TIRF microscopy.
(MOV)

S2 Movie Microtubule tracking in a control cell. Microtubules in control cell grow out to the periphery, briefly pause and shrink back into the cortex. The different colored numbers represent separate microtubules that were tracked by hand. S2 cell transfected with tRFP-α-tubulin. Images were acquired using TIRF microscopy.
(MOV)

S3 Movie Microtubule dynamics in a cell expressing Msps 3K3A where endogenous Msps is depleted. Microtubules in Msps 3K3A rescued cells spend more time growing, at a slower rate, than microtubules in control cells. S2 cell transfected with tRFP-α-tubulin and Msps GFP 3K3A. Images acquired using TIRF microscopy.
(MOV)

S1 Table Microtubule dynamics parameters. Colored cells represent a change that is statistically different from control. Pink indicates a decrease, with light pink representing a decrease ≤ 25% and dark pink representing a decrease of ≥ 25%. Green indicates an increase, with light green representing an increase ≤ 25% and dark green representing an increase of ≥ 25%. ^a^ Numbers in parentheses represent the number of cells and microtubules tracked, respectively. ^b^ Rescue frequency is calculated as the number of rescue events per second. Calculated as [total number of rescues] / [some time]. The time value is measured as only those times in which the microtubule can rescue. A rescue can only occur only when the microtubule is shrinking or pausing. Therefore, the time used in the calculation of rescue frequency would include only those periods when the MT is shrinking or pausing. ^c^ Catastrophe frequency is calculated as the number of catastrophe events per second. Calculated as [total number of catastrophes] / [some time]. The time value is measured as only those times in which the microtubule can catastrophe. A catastrophe can only occur only when the microtubule is growing or pausing. Therefore, the time used in the calculation of catastrophe frequency would include only those periods when the MT is growing or pausing. ^d^ Stall frequency is calculated as the number of stall events per second. Calculated as [total number of stalls] / [some time]. The time value is measured as only those times in which the microtubule can stall. A stall can only occur only when the microtubule is shrinking or growing. Therefore, the time used in the calculation of stall frequency would include only those periods when the MT is shrinking or growing. A threshold rate of 0.5um/sec was used. Microtubule movements under this threshold were not treated as a change in length.
(TIF)

## Abbreviations

Msps: Mini spindles
MAPs: microtubule-associated proteins
+TIP: microtubule plus end interacting protein
CA: Constitutively active
RNAi: RNA interference
ConA: concanavalin A
