# Supplementary figures and images for: TOG Proteins Are Spatially Regulated by Rac-GSK3β to Control Interphase Microtubule Dynamics
Source: PLoS One. 2015 Sep 25;10(9):e0138966. doi: 10.1371/journal.pone.0138966 (PMC4583408; doi:10.1371/journal.pone.0138966)

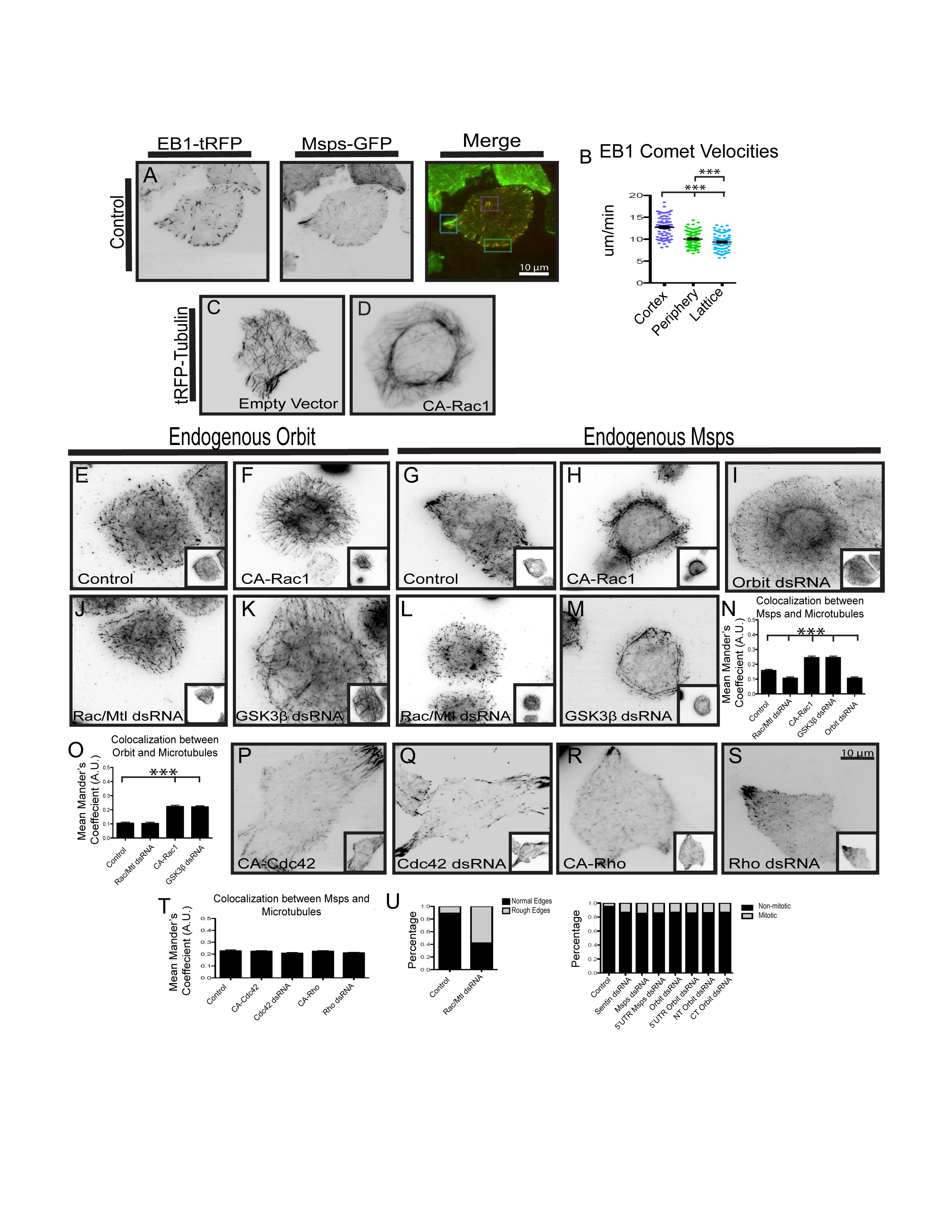

Supplement: S1 Fig — (A) EB1 and Msps expressing cells, merge image shows Msps in green and EB1 in red. The different colored boxes represent the different regions of the cells in which EB1 comets were tracked, purple represents the cortex of the cell, green represents the periphery and blue represents the lattice bound population. (B) Graph of the EB1 velocities in each population, colors correspond to the region of the cell. 20 cells were imaged and 5 comets per location in the cell were tracked results are from three experiments *** p<0.0001 (C) Control cell expressing a dual expression vector with tRFP- α-tubulin alone. (D) Cell expressing a dual expression vector with tRFP- α-tubulin and CA-Rac in the second site. (E) Endogenous Orbit and α-tubulin were stained in control cells and cells expressing CA-Rac1 (F). CA-Rac expressing cells were identified as those that were able to spread on glass coverslips without ConA. Rac1/Rac2/Mtl (J) or GSK3β (K) were knocked down with dsRNA and the cells stained for endogenous Orbit and α-tubulin. (G) Endogenous Msps and α-tubulin were stained in control cells (G) and cells expressing CA-Rac1 (H). Orbit (I), Rac1/Rac2/Mtl (L), or GSK3β (M) were knocked down with dsRNA in cell stained for endogenous Msps and α-tubulin. Tubulin images are shown as insets. (N-O) Changes in the co-localization of Msps (P) and Orbit (Q) with microtubules were measured using the Mander’s coefficient, n = 90 cells from three experiments. An increase indicates increased lattice binding and a decrease indicates decreased lattice binding. *** p<0.0001 (P-S) Msps-GFP is expressed in cells with a dual expression containing tRFP-α-tubulin and CA-Cdc42 (P) or CA-Rho (R). Cdc42 (Q) or Rho (S) were knocked down with dsRNA in cells expressing tRFP-α-tubulin. Tubulin images are shown as insets. (T) Changes in the co-localization of Msps and tubulin was measured using the Mander’s coefficient, n = 90 cells from three experiments. (U) Levels of depletion were measured using a functi [file pone.0138966.s001.tif]

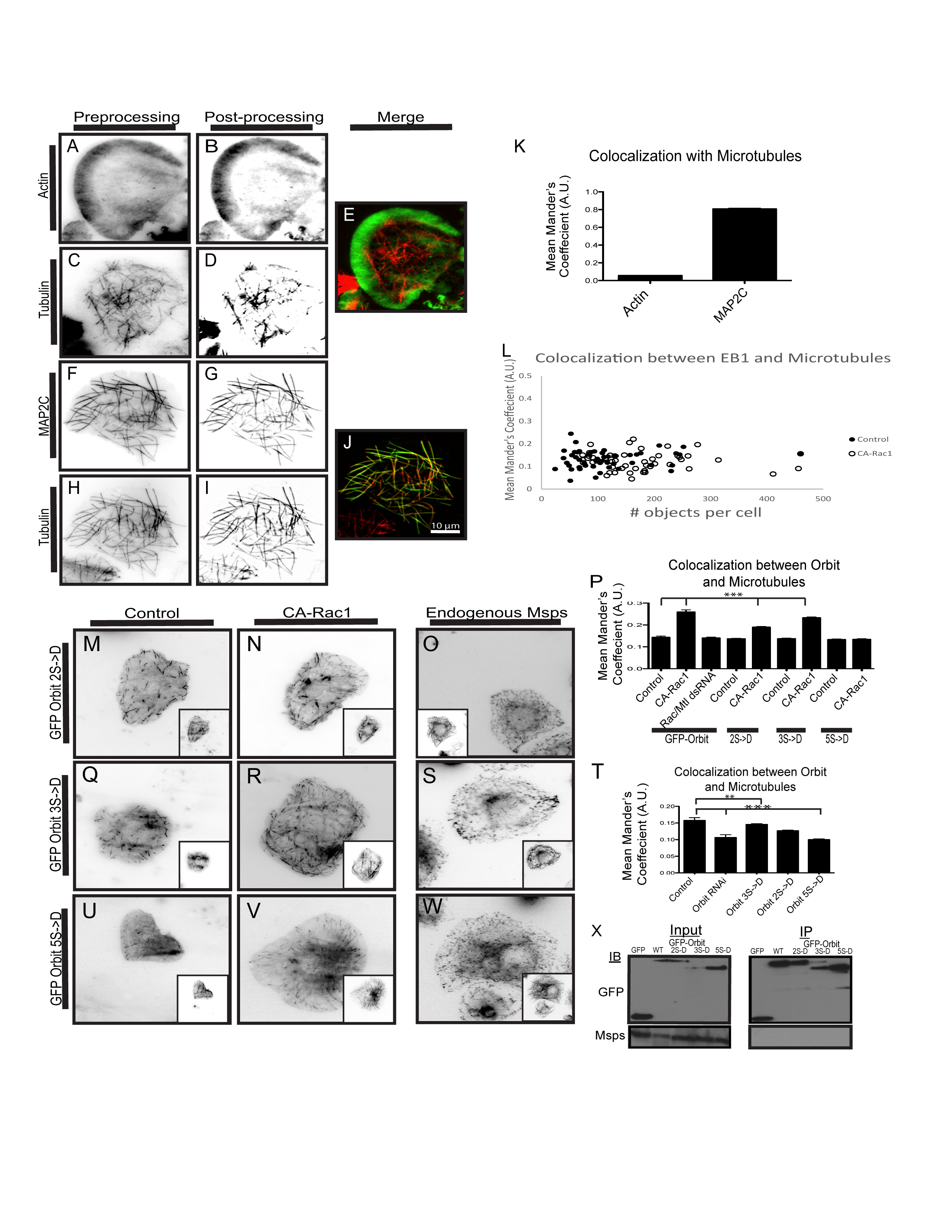

Supplement: S2 Fig — (A-J) Controls to test the efficiency of the Mander’s coefficient. Actin was used as a negative control. (A) GFP Actin expressing cell before processing and after subtraction of the background and despeckling (B). (C) tRFP- α-tubulin expressing cell before (C) and after processing (D). Merged image of post processed cell shows Actin in green and Msps in red. (F) MAP2C GFP expressing cell pre (F) and post (G) processing. tRFP- α-tubulin expressing cell pre (H) and post (I) processing, (J) Merged image shows MAP2C in green and tubulin in red. (K) Graph of the Mander’s coefficient of the two controls, N = 90 cells from three experiments. (L) Graph of the number of objects per cell (EB1 comets) versus the Mander’s coefficient of that cell. Images from both control (black dots) and CA-Rac1 expressing cells (white dots) were used. (M-O) GFP-Orbit 2S->D was expressed in cells with a dual expression vector containing tRFP-α-tubulin alone (M) or with CA-Rac1 (N). (O) Endogenous Msps and α-tubulin were stained in cells transfected with 2S->D. (Q-S) GFP-Orbit 3S->D is expressed in cells with a dual expression vector containing α-tubulin-tRFP alone (Q) or with CA-Rac1 (R). (S) Endogenous Msps and α-tubulin were stained in cells transfected with 2S->D. (U-W) GFP-Orbit 5S->D was expressed in cells with a dual expression vector containing tRFP-α-tubulin alone (U) or with CA-Rac1 (V). (W) Endogenous Msps and α-tubulin were stained in cells transfected with 5S->D. Tubulin images are shown as insets. (P and T) Changes in co-localization of Orbit (P) and endogenous Msps (T) were measured using the Mander’s coefficient, n = 90 cells from two (endogenous Msps) or three (GFP-Orbit) experiments. *** p<0.0001. (X) Msps cannot coimmunoprecipitate with phosphomemetic mutants of Orbit. Immunoprecipitations were performed from cells depleted of endogenous Orbit using dsRNA targeting the 5'UTR of the gene and rescued with the indicated GFP-tagged Orbit constructs. (TIF) [file pone.0138966.s002.tif]

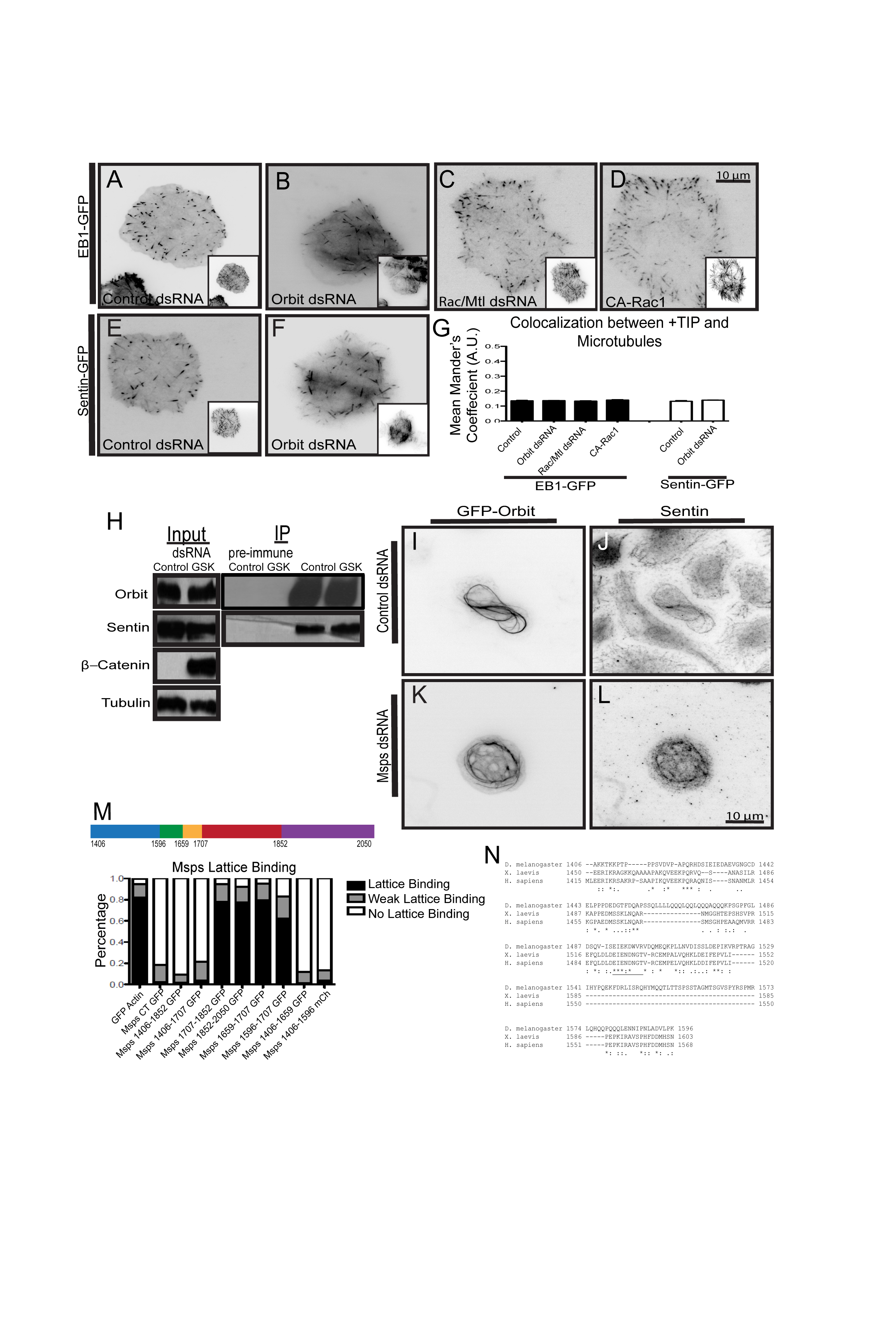

Supplement: S3 Fig — (A-D) EB1-GFP was expressed in cells with a dual expression vector containing tRFP-α-tubulin alone (A) or CA-Rac1 (D) and also in cells with Orbit (B) or Rac1/Rac2/Mtl depletion (C). (E-F) Sentin-GFP was expressed in cells with tRFP- α-tubulin with control (E) or Orbit depletion (F) Tubulin images are shown as insets. (G) Changes in co-localization of EB1 and Sentin were measured using the Mander’s coefficient, n = 90 cells from three experiments. (H) Immunoprecipitation of Sentin for Orbit. Pre-immune serum was taken from rabbits prior to injection with the Orbit antigen. GSK3β depletion was assessed using β-catenin levels, with tubulin as a loading control. (I-L) GFP-Orbit was overexpressed in cells stained for endogenous Sentin and α-tubulin with control (I-J) or Msps (K-L) depletion. (M) Schematic of the C-terminus of Msps colored by regions of predicted secondary structure. The extent of Msps binding to the microtubule lattice in cells expressing different Msps C-terminal constructs. Different constructs were scored as lattice binding, weak lattice binding, or no lattice binding. (N) CLUSTAL alignment of the Msps C-terminus with higher eukaryote homologs, Xenopus laevis XMAP215 and Homo sapiens ch-TOG. The underline denotes a region of sequence conservation. (TIF) [file pone.0138966.s003.tif]

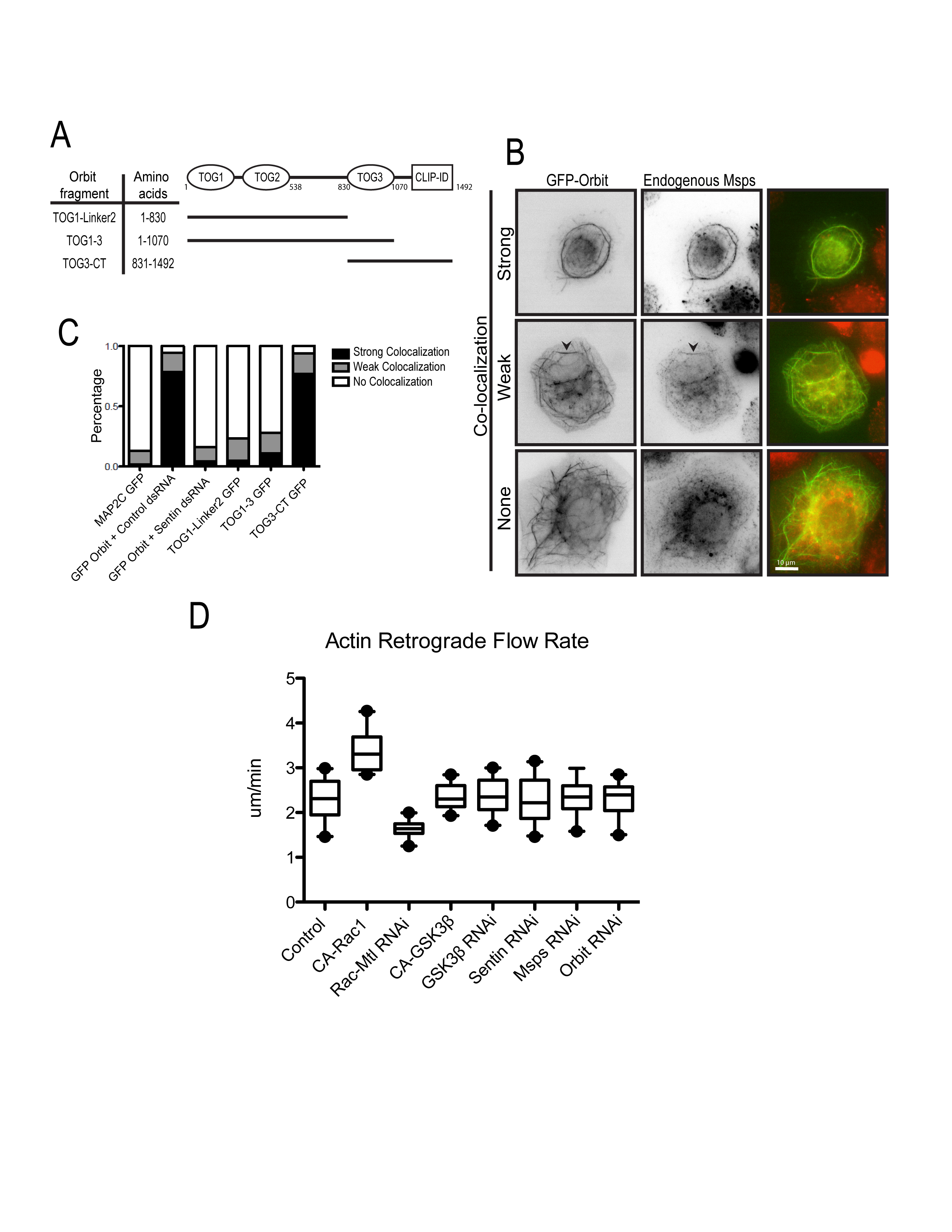

Supplement: S4 Fig — (A) A schematic of the domain structure of Orbit and different fragments expressed in cells. (B) Examples of Strong, Weak and No colocalization of Msps with overexpressed Orbit. (C) Msps lattice association in cells expressing the different constructs was scored as strong, weak or no colocalization with lattice bound Orbit. (D) Rates of actin retrograde flow is altered only by changes in Rac activity. Actin retrograde flow was measured using kymographs of actin particle movement from the cell periphery to the cortex, n = 20 cells from three experiments. *** p<0.0001. (TIF) [file pone.0138966.s004.tif]

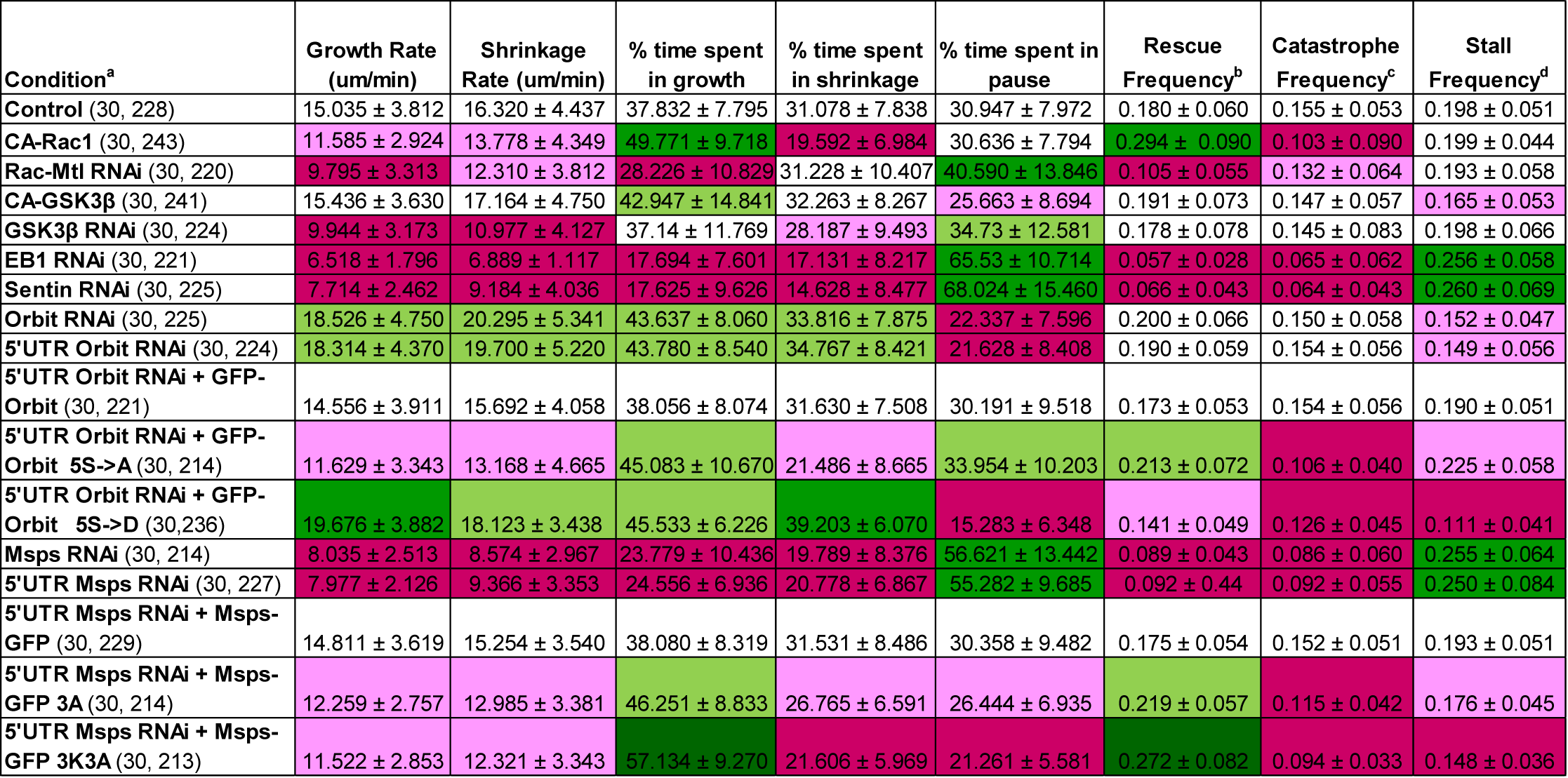

Supplement: S1 Table — Colored cells represent a change that is statistically different from control. Pink indicates a decrease, with light pink representing a decrease ≤ 25% and dark pink representing a decrease of ≥ 25%. Green indicates an increase, with light green representing an increase ≤ 25% and dark green representing an increase of ≥ 25%. a Numbers in parentheses represent the number of cells and microtubules tracked, respectively. b Rescue frequency is calculated as the number of rescue events per second. Calculated as [total number of rescues] / [some time]. The time value is measured as only those times in which the microtubule can rescue. A rescue can only occur only when the microtubule is shrinking or pausing. Therefore, the time used in the calculation of rescue frequency would include only those periods when the MT is shrinking or pausing. c Catastrophe frequency is calculated as the number of catastrophe events per second. Calculated as [total number of catastrophes] / [some time]. The time value is measured as only those times in which the microtubule can catastrophe. A catastrophe can only occur only when the microtubule is growing or pausing. Therefore, the time used in the calculation of catastrophe frequency would include only those periods when the MT is growing or pausing. d Stall frequency is calculated as the number of stall events per second. Calculated as [total number of stalls] / [some time]. The time value is measured as only those times in which the microtubule can stall. A stall can only occur only when the microtubule is shrinking or growing. Therefore, the time used in the calculation of stall frequency would include only those periods when the MT is shrinking or growing. A threshold rate of 0.5um/sec was used. Microtubule movements under this threshold were not treated as a change in length. (TIF) [file pone.0138966.s008.tif]
